# Functional Water Networks in Fully Hydrated Photosystem II

**DOI:** 10.1021/jacs.2c09121

**Published:** 2022-11-22

**Authors:** Abhishek Sirohiwal, Dimitrios A. Pantazis

**Affiliations:** Max-Planck-Institut für Kohlenforschung, Kaiser-Wilhelm-Platz 1, 45470 Mülheim an der Ruhr, Germany

## Abstract

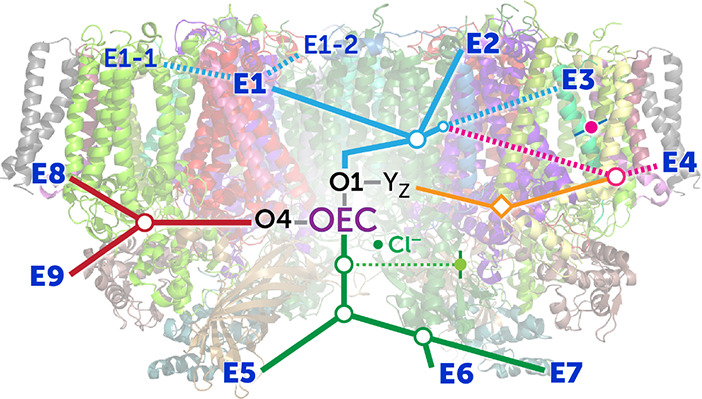

Water channels and networks within photosystem II (PSII) of oxygenic photosynthesis are critical for enzyme structure and function. They control substrate delivery to the oxygen-evolving center and mediate proton transfer at both the oxidative and reductive endpoints. Current views on PSII hydration are derived from protein crystallography, but structural information may be compromised by sample dehydration and technical limitations. Here, we simulate the physiological hydration structure of a cyanobacterial PSII model following a thorough hydration procedure and large-scale unconstrained all-atom molecular dynamics enabled by massively parallel simulations. We show that crystallographic models of PSII are moderately to severely dehydrated and that this problem is particularly acute for models derived from X-ray free electron laser (XFEL) serial femtosecond crystallography. We present a fully hydrated representation of cyanobacterial PSII and map all water channels, both static and dynamic, associated with the electron donor and acceptor sides. Among them, we describe a series of transient channels and the attendant conformational gating role of protein components. On the acceptor side, we characterize a channel system that is absent from existing crystallographic models but is likely functionally important for the reduction of the terminal electron acceptor plastoquinone Q_B_. The results of the present work build a foundation for properly (re)evaluating crystallographic models and for eliciting new insights into PSII structure and function.

## 1 Introduction

Water actively determines the internal structure of enzymes, mediates proton transfer, regulates redox properties, and even participates as reactant in chemical transformations. Nowhere is this better exemplified than in photosystem II (PSII), the membrane-embedded pigment–protein complex that harnesses sunlight to drive water oxidation and plastoquinone reduction.^1−10^ Every chemical transformation in PSII, from the four-electron water oxidation at the oxygen-evolving complex (OEC) to the two-electron reduction of the mobile electron carrier plastoquinone, is intimately coupled to water management and proton transfer. Thus, atomic-level elucidation of enzymatic functions relies on precise understanding of water channels and networks within PSII.^11−37^

Crystallographic studies provide crucial structural information in this respect.^38−43^ To the extreme, apparent alterations of crystallographic water positions in time-resolved studies have even been used as basis for speculations on mechanistic scenarios.^33,44−47^ However, the information content of crystallographic models of PSII is constrained by more than medium resolution and inherent technical limitations in locating water molecules or identifying their dynamics. Dehydration is employed in membrane protein crystallography to improve packing and reduce mosaicity, thus facilitating structure determinations and enhancing resolution.^48−55^ On the other hand, loss of water affects the reaction center absorption profiles, inhibits the water oxidation cycle at the OEC, hampers electron transfer among plastoquinones, and compromises overall oxygen evolution activity.^56−61^ Despite impairment of physiological function, post-crystallization dehydration is not undesirable because it is a technically beneficial procedure that opened the way toward high-resolution structures of PSII. Nevertheless, the dehydrated sample is, by definition, insufficiently representative of the physiological state even if the global integrity of the enzyme is maintained. The consequences can be far-reaching if reduction in water content leads to inaccurate representation of functionally critical regions such as the active sites of water oxidation and plastoquinone reduction.

**Figure 1:**
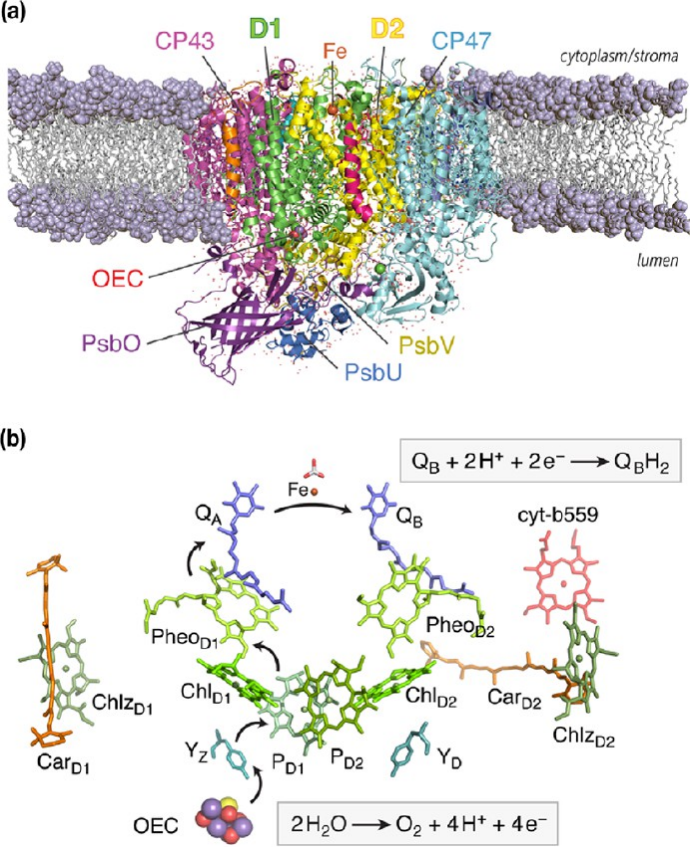
(a) Model of the membrane-embedded PSII monomer. Key protein subunits are labeled. The location of the oxygen-evolving complex and the non-heme iron are indicated. The PsbO, PsbU, and PsbV proteins cap cyanobacterial PSII on the lumenal side of the membrane. (b) Redox-active cofactors in the core proteins of PSII and the two reactions accomplished at the oxidative and reductive termini. The arrows indicate the normal flow of electrons.

In this work, we show that all of the above issues arise in available crystallographic models of PSII. To recover missing information on hydration, we construct a membrane-embedded model of PSII (Figure 1) and employ a multistage hydration procedure coupled to unbiased molecular dynamics (MD) simulations on high-performance graphics processing units. This enables us to reconstruct an approximated physiological hydration structure of cyanobacterial PSII. On this basis, we evaluate the hydration state of crystallographic models. We quantify the extent of dehydration in conventional (synchrotron crystallography) and more recent X-ray free electron laser serial femtosecond crystallography (XFEL-SFX) models, and we show that the latter are drastically dehydrated, not only in a global sense but also internally around the OEC and on the acceptor side of the enzyme. Subsequently, we map all water channels associated with the oxidative and reductive endpoints of PSII. Thanks to the employed simulation time scale, we are able to document new water channel systems and entry/exit points, some of them transiently formed, and describe how they are gated by protein conformational changes. Finally, we characterize a channel system in the stromal side of PSII that is absent from crystallographic models but has an obvious functional role, thus establishing the physiological hydration state of the acceptor side and offering a structural basis for discussing the hydration of the non-heme iron site and the mechanism of plastoquinone reduction.

## 2 Results and Discussion

### 2.1 Hydration and Molecular Dynamics Simulations

The diffusion time scale of bulk water to internal cavities can be very long and requires extensive sampling of protein conformations. Therefore, conventional MD simulations need to be performed for longer than typical time scales to accumulate information about the dynamics of buried water molecules. The calculations scale unfavorably with the increasing size of the system, especially for the complete membrane-embedded PSII model. Alternative strategies such as the three-dimensional reference interaction site model (3D-RISM) or integration of the Monte Carlo (MC) method with molecular dynamics (for instance, the MC/MD module implemented in AMBER) have shown tremendous capabilities in predicting the hydration content or solvent-accessible sites inside inhomogeneous environments. In this work, we employed the 3D-RISM^62,63^ and MC/MD^64^ techniques to hydrate internal cavities of PSII prior to production simulations. 3D-RISM is an integral statistical theory of molecular liquids, which can be used to obtain the 3D density distribution of solvent around a solute, while the MC/MD method is based on the integration of the Metropolis Monte Carlo translational movement of water from the bulk to internal cavities during the MD simulation.

The PSII crystal structure by Umena *et al.*(41) (PDB ID: 3WU2, monomer A) was used as the starting point. We placed emphasis on completing all structural details that are missing from the crystallographic structure in order to make our computational model as representative of the natural system as possible. The prepared PSII structure was embedded in a large patch of lipid bilayer. In addition, a larger than typical dimension of the water box was used along the membrane normal to eliminate any artificial protein–protein interactions during the MD simulations. The setup is fully described in the Supporting Information.

**Figure 2:**
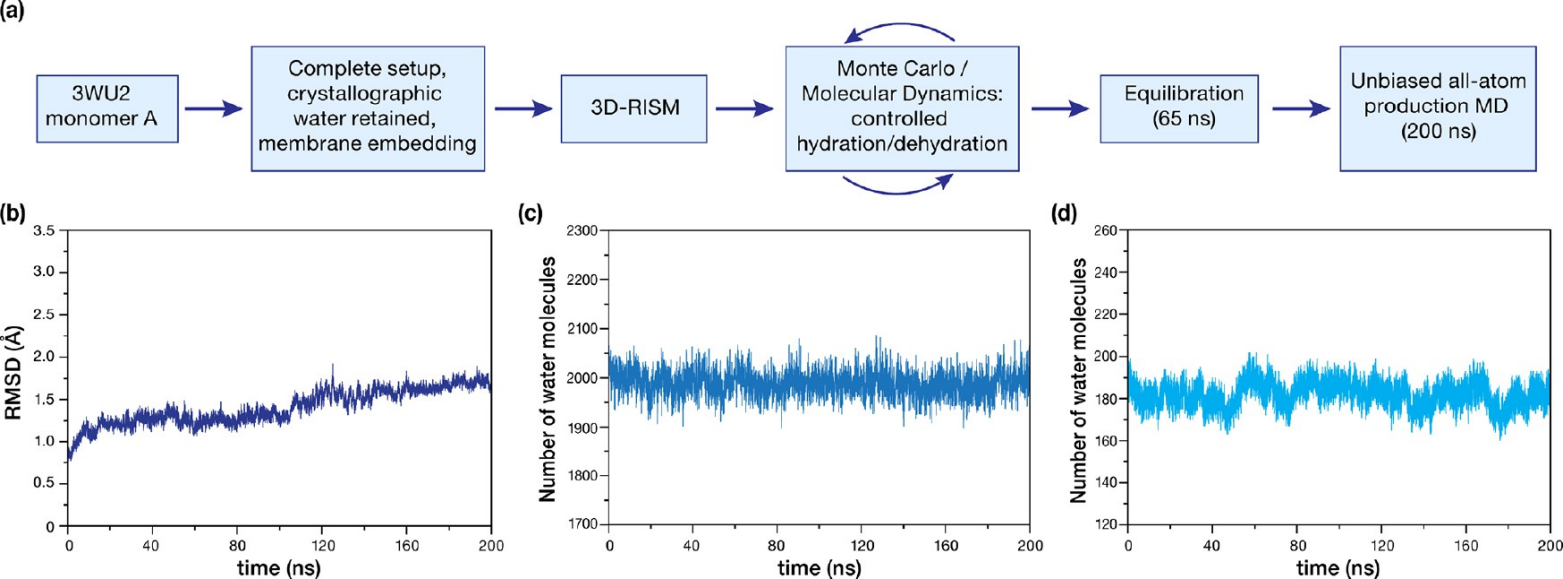
(a) Flow-chart describing the simulation procedure employed in the current work. (b) Time evolution of root mean square deviation (RMSD) of Cα atoms of the PSII monomer during production simulations (data recorded every 2 ps, excluding the highly flexible loops). (c) Time evolution of the hydration content of the PSII monomer. All water molecules in close contact (∼2 Å) with PSII were selected in the counting (data recorded every 50 ps, hydration content around the highly flexible loops not considered). (d) Time evolution of water content within a sphere of radius 20 Å centered around the OEC (data recorded every 2 ps). Corresponding datasets that include the water content associated with the highly flexible loops are provided in the Supporting Information.

Given that major effort is devoted to properly hydrate the system before initiating production simulations, a thorough equilibration procedure is followed to ensure proper hydration. As a first step, we kept all crystallographically resolved (1506) water molecules. In a second step, we used the 3D-RISM technique coupled with the *placevent* module^65^ to further identify explicit water positions based on the 3D distribution density. A total of 5404 water molecules were predicted using the latter procedure. The majority of waters were found on the protein–bulk interface, but significant water content was also placed in internal protein cavities. Importantly, we found 32 new water positions within 20 Å around the OEC. This single point 3D-RISM calculation was performed on the crystallographic configuration of the PSII protein complex. However, to fully reverse the dehydration of the crystallographic model, it requires a dynamic treatment of hydration on the computational side, that is, rehydrating in conjunction with time evolution of the system. Following a thorough structural relaxation procedure, we thus performed MC/MD calculations to fill or vacate internal cavities based on energetic criteria. Complete algorithmic details are described by Gilson and coworkers.^64^ The schematic workflow of the complete hydration procedure is shown in Figure 2a. Thereafter, we conducted MD simulations for 65 ns to equilibrate the complete system, lipid-bilayer, and specifically to allow the newly added water molecules to reach equilibrium with the bulk. Once the system was completely hydrated and fully equilibrated, we initiated 200 ns of production MD simulation for final data acquisition (Figure 2b). Complete technical details on the hydration protocol, equilibration, and production MD simulations are provided in the Supporting Information.

The total hydration content of the PSII monomer is depicted in Figure 2c. All water molecules in close contact (∼2 Å) with PSII were selected in the counting. We observe that the total hydration content of the enzyme remains stable during the simulation. The PSII monomer can hold a minimum of 1897 and a maximum of 2087 number of water molecules, which depends on the conformational state of the protein. In addition, we observe that the conformational flexibility of the protein influences the local architecture of water channels. As a demonstration of this point, we chose a spherical region centered around the OEC with a radius of 20 Å, a distance that covers the maximum internal protein volume while completely avoiding the bulk and transmembrane regions. We observe that the time evolution of the hydration content within this volume remains stable (Figure 2d), even though discrete fluctuations are observed. These reflect the dependence of water content on protein flexibility, manifested as conformational gating of water channels by inter- and intra-subunit salt bridges, side chain rotation of amino acids, and the conformational dynamics of C-terminal residues. An atomistic description of such elements is discussed in the following.

### 2.2 Comparison with Crystallographic Models

Although water is obviously lost by dehydration procedures,^48,49^ it is hard to estimate the actual extent of water content reduction. In Table 1, we provide a view into this problem by comparing the number of explicit water molecules resolved in various crystallographic models^33,41−47,66^ and two recent cryo-electron microscopy (cryo-EM) structures^67,68^ with the results of our MD simulations. This can serve as an indicator for the wetness of the system. Deviation from the MD hydration content is not a strictly quantitative measure of sample dehydration because the difference from the MD reference cannot be uniquely attributed to the real absence of water. Experimental resolution limitations and incomplete correspondence between crystallographic and computational models introduce ill-defined uncertainties. Nevertheless, the comparison does provide a meaningful, if only semi-quantitative, indication of how far the crystallographic models are from a fully hydrated state.

**Table 1:** Total Number of Water Molecules and Hydration Content Within 20 Å from the Oxygen Evolving Complex (OEC) Derived from the Molecular Dynamics Simulations of the Fully Hydrated PSII Monomer Compared to the Number of Water Molecules Present in Selected Crystallographic Models of PSII from Synchrotron (SX) and Free Electron Laser (XFEL) Sources and from Cryo-Electron Microscopy Studies

			total water count^a^			20 Å - OEC	
	expt. type	resolution (Å)	monomer I^b^	monomer II^c^	sum	I	II
MD			1989			182^d^	
5B66^43^	SX	1.85	1957	1968	3925	185	179
5B5E^43^	SX	1.87	1810	1814	3624	185	178
3WU2^41^	SX	1.90	1506	1378	2884	184	178
4UB6^42^	SX	1.95	1377	1213	2590	176	179
7D1T^67^	Cryo-EM	1.95	1222	1210	2432	174	174
4IL6^66^ (Sr)^e^	SX	2.10	1079	958	2037	169	166
6W1O^47^	XFEL (RT)	2.08	1081	956	2037	156	156
6DHE^46^^f^	XFEL (RT)	2.05	1082	939	2021	165	155
6DHP^46^^g^	XFEL (RT)	2.04	1072	946	2018	162	159
6DHF^46^	XFEL (RT)	2.08	1081	936	2017	165	159
7RF1^33^	XFEL (RT)	1.89	1016	944	1960	165	165
5GTI^44^	XFEL	2.50	945	966	1911	162	163
5GTH^44^	XFEL	2.50	881	900	1781	158	162
5WS5^44^	XFEL	2.35	809	817	1626	165	166
7N8O^68^^h^	Cryo-EM	1.93	619	617	1236	156	156
5TIS^45^	XFEL (RT)	2.25	571	608	1179	116	124

a Average number of water molecules, data recorded every 50 ps. Water around the highly flexible regions (disordered in many crystallographic models) is excluded from the water count. The maximum and minimum total numbers of water molecules from the MD simulations are 2087 and 1897, respectively. If the flexible regions are included, then the average, maximum, and minimum numbers of water molecules around the complete PSII model from the MD simulations are 2050, 2153, and 1948, respectively. Water molecules with fractional occupancy in the experimental structures are counted as a single unit.

b Chains with an upper letter case.

c Chains with a lower letter case.

d Maximum and minimum numbers of waters are 202 and 160, respectively.

e Strontium-substituted OEC.

f S_1_-enriched sample.

g S_0_-enriched sample.

h Mesophilic cyanobacterium *Synechocystis sp.* PCC 6803.

The number of resolved water molecules varies greatly among different crystallographic models, and non-negligible differences in water content may also exist between PSII monomers within the same crystal structure. The model of Tanaka et al.^43^ (PDB ID: 5B66) exhibits the highest water content among all crystallographic models and is remarkably close in absolute terms to the water content predicted by the MD simulations. Importantly, this also implies that the MD simulations did not artificially result in overhydration of the computational model. Other crystallographic models derived from conventional synchrotron setups have lower water count. Structural models derived from XFEL-based crystallographic studies are the least hydrated. The model reported by Young et al.^45^ (PDB ID: 5TIS) has the lowest water count, with apparent water content merely one third of that in the Tanaka et al.^43^ model or in the present MD model, even though it does not have nominally the lowest resolution among XFEL models. The two cryo-EM structures are considerably different from each other in terms of water count. 7D1T^67^ appears more hydrated than any XFEL model but still does not compare favorably with synchrotron-based crystallographic models.

The total hydration content is a global indicator, but information regarding internal water is more discerning as it relates to conserved channel systems. To probe this more specifically, we counted water molecules within a sphere of 20 Å radius centered at the OEC. The average number of water molecules within the OEC-centered spherical region from the MD simulations is 182 (the range spans a maximum of 202 and a minimum of 160 water molecules, which demonstrates the extent to which protein conformation and flexibility modulate hydration). Most of the conventional crystallographic models are in very good agreement with the MD simulations. Interestingly, the crystallographic models by Tanaka et al.^43^ (5B66) and by Umena et al.^41^ (3WU2), which showed large differences in the total hydration content (451 and 590 waters in the case of monomers I and II, respectively), yield nearly the same number of waters within the OEC-centered sphere. This shows that most unresolved water molecules in the 3WU2 model are close to the protein periphery and not in the interior.

The most interesting observation concerns again the XFEL-based crystallographic models. These present considerably lower water content even within the *internal* OEC-centered sphere, with the 5TIS model showing an unreasonably small number of water molecules. The low manifest water content at the donor side of PSII in XFEL-SFX models may be due to dehydration of the samples or to resolution limitations. However, the fact that the recent 7RF1 model by Hussein et al.^33^ is reported to have an effective resolution rivaling the best synchrotron crystallographic models, yet suffers from a similarly low water count around the OEC as other XFEL-derived models, suggests that the diminished water count reflects a real departure of the samples from the physiological hydration state.

These results show that currently available XFEL-SFX models of PSII present an inadequate picture of the hydration state of the system, as a consequence of the severe dehydration of the samples. Crucially, these problems extend deep into the protein and even within the proximity of the OEC. Therefore, these models offer a precarious structural basis for inferences regarding the atomistic nature of water channels and may not be sufficiently reliable for extracting mechanistic information based on observed movements of water around the OEC site.

### 2.3 Architecture of Water Channels Around the OEC

**Figure 3:**
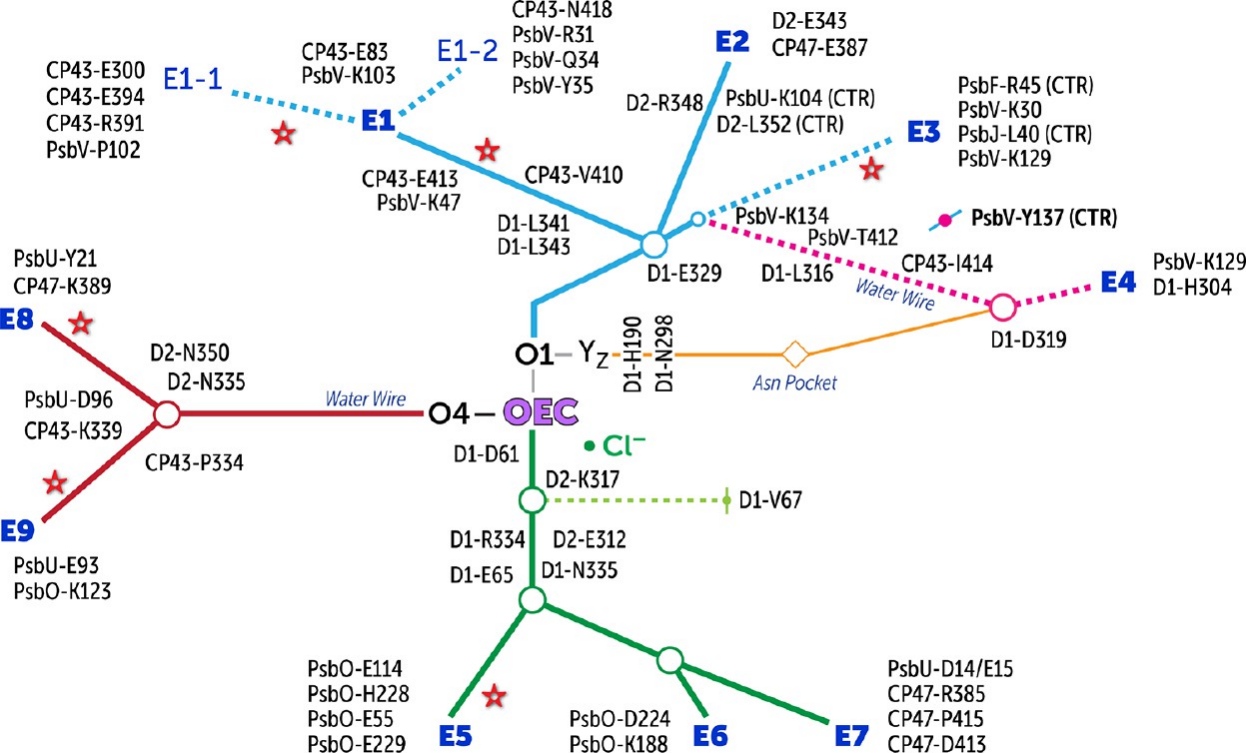
Identification of water channels leading from the bulk toward the OEC, along with the proposal for the proton exit pathway associated with Y_Z_. O1 (blue), O4 (red), and Cl1 (green) channel systems are colored uniquely for presentation purposes. Asterisks indicate positions of DMSO molecules found in the 5B66 crystallographic model. Dotted lines imply the transient nature of respective water channels due to protein conformational dynamics.

In addition to the high oxidation potential generated at the reaction center, efficient catalytic action of the oxygen evolving complex requires channels for substrate water delivery, proton release, and efficient diffusion of dioxygen. In line with previous studies, our results show three major water channel systems in the lumenal side of PSII connecting the bulk phase with the OEC (see Figure 3). We follow recent convention and name them after the closest terminating atom of the OEC cluster, i.e., O1, O4, and Cl1 channel systems (the term “channel system” implies that they all consist of more than one water chain or branch). These approximately correspond to the “narrow”, “large”, and “broad” channels discussed in the past literature; for channel nomenclatures used in various studies, we refer the reader to the excellent summary by Hussein et al.^33^ These systems remain hydrated throughout the simulation and no significant change in hydration content is observed within 20 Å around the OEC (Figure 2d). However, differences exist in terms of their length, width, tributaries (contributing minor channels that terminate at the protein–bulk interface), and presence of protein conformation-dependent transient channels.^14,16,20,25,27,30,69^ We label the minor branches that contribute to the three major channel systems as E1–E9 (see Figure 3). In the following, we describe the key characteristics of each system.

#### 2.3.1 O1 Water Channel System

**Figure 4:**
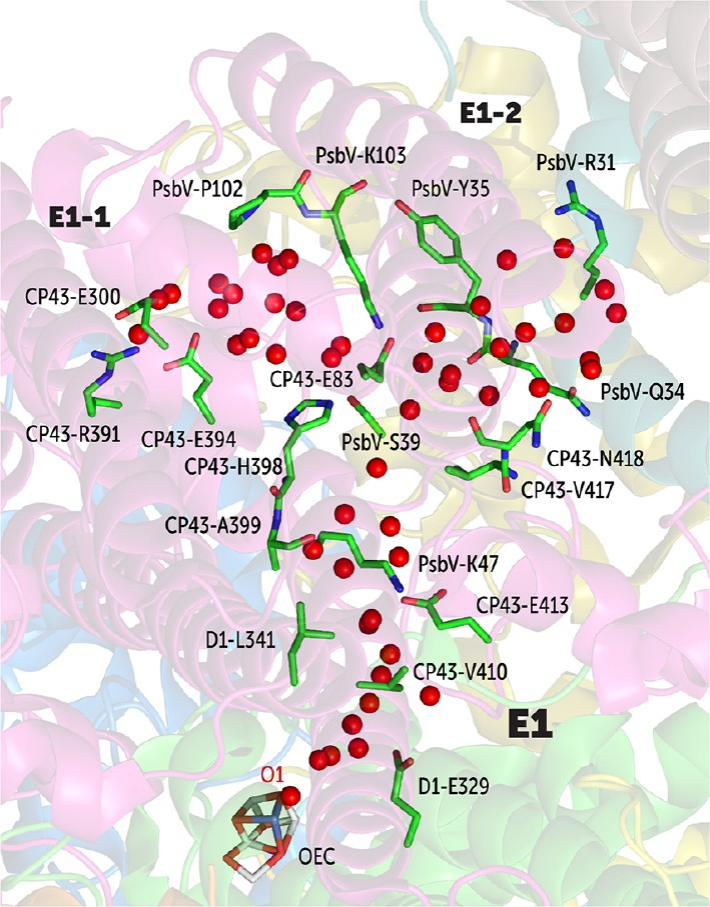
Depiction of the E1 branch of the O1 channel system.

**Figure 5:**
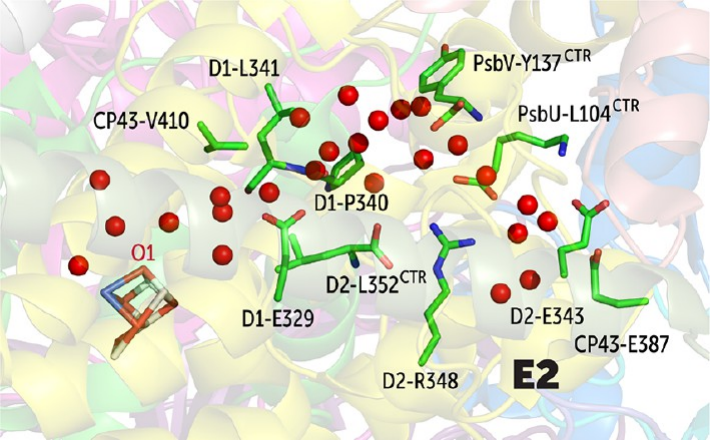
Depiction of the E2 branch of the O1 channel system. The superscript CTR denotes C-terminal residues.

Channel system O1 contains in total four minor contributing channels (E1–E4, Figures 3–5), of which E1 and E2 remain hydrated and active in water exchange with the bulk throughout the simulation, whereas hydration of the other two channels E3 and E4 is dynamic and conformation-dependent. Specifically, which one is active depends on the conformational dynamics of PsbV-Tyr137, a C-terminal residue of the extrinsic PsbV protein. All four contributing channels merge at a region near D1-Glu329. The O1 channel system incorporates an internal water network associated with the redox-active D1-Tyr161 (Y_Z_) residue that appears to connect with E4 but does not exchange waters with the bulk during our simulations.

The E1 channel is located at the interface between CP43 and PsbV. We observe that water can enter or exit E1 through two small water gates, labeled E1-1 and E1-2. In the case of E1-1, the incoming water makes first contact with the CP43-Glu300, CP43-Glu394, CP43-Arg391, and PsbV-Pro102 residues, whereas in the case of E1-2, contact is made in a region surrounded by PsbV-Arg31, PsbV-Gln34, PsbV-Tyr35, and CP43-Asn418 (Figure 4). Water molecules through both E1-1 and E1-2 merge at a junction consisting of a CP43-Glu83/PsbV-Lys103 salt-bridge and then pass through CP43-His398, CP43-Ala399, PsbV-Ser39, PsbV-Lys47 and CP43-Glu413 salt bridge, D1-Leu341, and CP43-Val410 to merge with the other channels (E2, E3, and E4) near D1-Glu329. The E1 is wider than the other contributors to the O1 channel system, and therefore, it contains more water molecules in the cross section. In this regard, it is also important to note that DMSO (dimethyl sulfoxide, used as cryoprotectant) is found inside this channel in the 5B66 crystallographic model^43^ (Figure S5) and lies sufficiently close to the OEC, with its further penetration blocked by D1-Leu341, CP43-Val410, CP43-Leu401, and D1-Leu343. It is also noted that a glycerol molecule in the model by Umena et al.^41^ is found to enter PSII through exactly the same cavity.

Water molecules in the E2 branch exchange through the interface created by residues D2-Glu343, CP47-Glu387, PsbU-Lys104 (C-terminal), D2-Leu352 (C-terminal), PsbV-Tyr137 (C-terminal), and D2-Arg348 (Figure 5). The C-terminal carboxylate groups of PsbU-Lys104, D2-Arg348, and D2-Leu352 form a triple salt-bridge-like interaction. The water in this channel also merges with other channels near D1-Glu329. It should be pointed out that the spatial region corresponding to this particular entry point overlaps with one of the entry points (E8) of the O4 water channel to be discussed in the following, i.e., the two channel systems are not entirely isolated from each other close to the interface.

**Figure 6:**
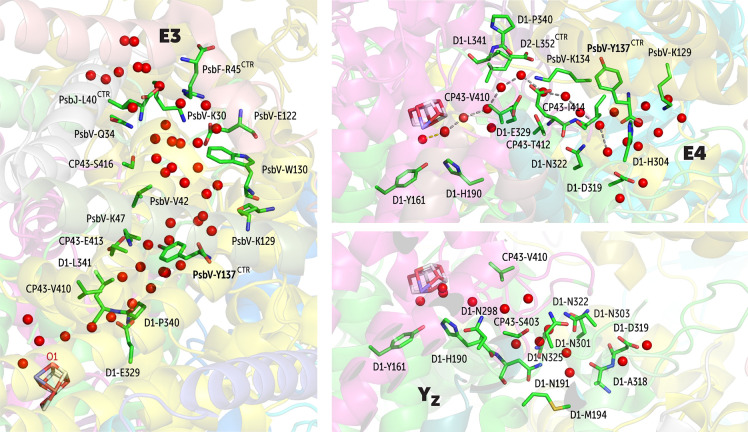
Depiction of the E3 and E4 branches of the O1 channel system, along with the Y_Z_ water network that converges to E4 close to the D1-Asp319 residue.

The transient nature of the other two entry points, E3 and E4, is dependent on the conformational dynamics of PsbV-Tyr137 (C-terminal). In the case of E3, the incoming water first makes contact with the PsbF-Arg45 (C-terminal), PsbV-Lys30, and PsbJ-Leu40 (C-terminal) (Figure 6). The C-terminal carboxylate of PsbJ-Leu40 creates a triple salt-bridge-like interaction with the PsbV-Lys30 and PsbF-Arg45. Upon entry to this channel, the water passes through PsbV-Glu122, CP43-Ser416, PsbV-Trp130, PsbV-Lys129, and PsbV-Tyr137 and later merges with other channels at D1-Glu329. In the case of the E4 channel, we observed the formation of a linear water wire that is highly ordered so it might also serve in proton translocation (Figure 6). The incoming water in this case makes first contact with the PsbV-Tyr137 (C-terminal), PsbV-Lys129, and then with D1-Asp319 and D1-His304. Thereafter, the water passes through CP43-Ile414, CP43-Thr412, CP43-Glu413, and PsbV-Lys134 and merges with the other channels at D1-Glu329.

**Figure 7:**
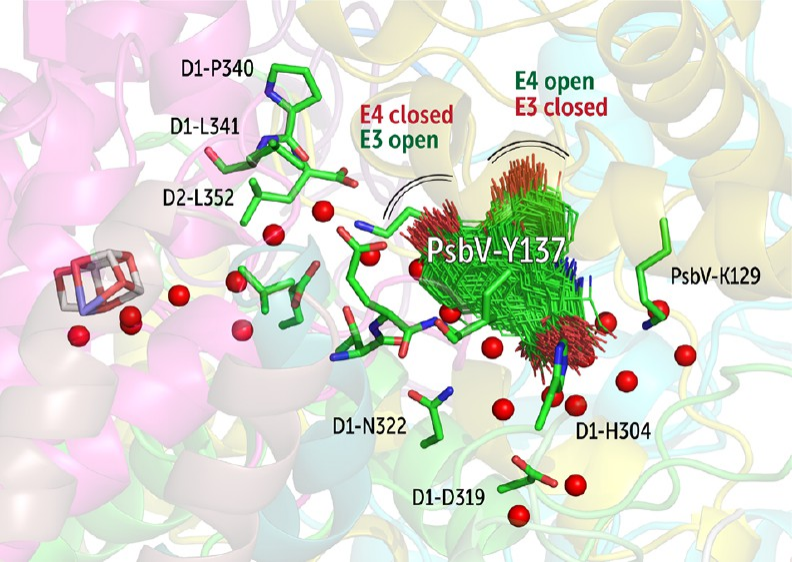
Role of the PsbV-Tyr137 residue in gating the E3 and E4 channels by adopting two distinct orientations.

PsbV-Tyr137 populates two conformational states during the MD simulations, only one of which allows each one of the channels to become active (Figure 7), therefore functioning as a switch. This point regarding the conformational dependence of the activation of E3 and E4 is also reflected in the most hydrated crystal structure by Tanaka et al.,^43^ where only the E3 water channel is present, whereas E4 is absent (dehydrated, Figure S6). The hydration and formation of active water transport through E4 may have mechanistic consequences for the function of the OEC because the end-point of the E4 channel toward the bulk interacts with D1-Asp319, which is connected to the Y_Z_(D1-Tyr161)-His190-Asn298 triad through an extensive hydrogen bond network of waters and residues.

It is important to note that the Y_Z_ water system does not exchange waters with the bulk. The water molecules inside this channel are retained throughout the MD simulations, as in previous reports^25^ and thus may only serve to establish a robust hydrogen bonding network for proton translocation upon Y_Z_ oxidation, presumably during one of the S-state transitions.^70,71^ A peculiar part of the Y_Z_ water network is a pocket that is almost exclusively lined by asparagine residues (indicated as “Asn pocket” in Figure 3).^70^ It has been speculated that the associated hydrogen bond network might serve for proton storage, stabilizing the proton via extensive hydrogen bonding,^25,72^ potentially assisted by Asn tautomerization.^70^ If a proton could also exit via the Y_Z_ branch,^70^ then this may occur only when the E4 terminal is active, since this enables entry and exit of water necessary for proton discharge into the bulk. In this scenario, by controlling the state of E4, PsbV-Tyr137 may play a role in proton transfer from Y_Z_. A recent site-directed mutagenesis study by Xiao et al.^73^ showed the importance the PsbV-Tyr137 in regulating oxygen evolution and stabilizing other extrinsic proteins. Based on their results, it was proposed that PsbV-Tyr137 is crucial for the stable binding of the PsbV protein and for facilitating proton egress from Y_Z_. Apart from PsbV-Tyr137, we also observe that PsbV-Lys134 makes an important salt-bridge interaction with the D1-Glu329 and D2-Leu352 (C-terminal). This particular region is important as all small tributaries of the O1 channel system merge here. Therefore, we conclude that the extrinsic PsbV protein is critical for the proper functioning and regulation of the entire O1 water channel system.

Recent site-directed mutagenesis experiments on the terminal residues of the E4 channels further supports the presents finding. Zhu et al. observed significantly decreased oxygen evolving activity (63–91% compared to the wild-type) in samples with mutations of D1-Arg323, D1-Asn322, D1-Asp319, and D1-His304.^74^ These residues lie at the terminus of the E4 channel. In addition, it was found that the PsbV protein was lost in these mutants.^74^ Overall, these experimental observations combined with the present computational results further strengthen the argument regarding the role of these key residues of the E4 channel in proton egress from the OEC, and they support the role of PsbV in regulating oxygen evolution.

#### 2.3.2 Cl1 Water Channel System

**Figure 8:**
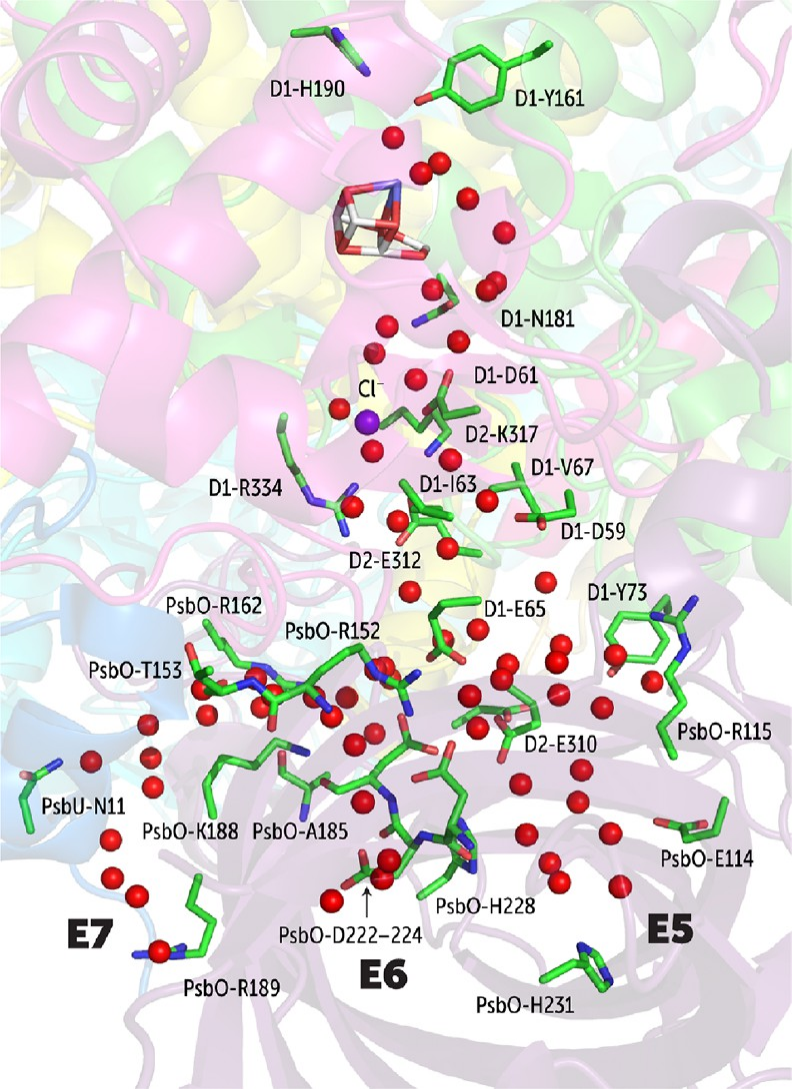
Depiction of the E5, E6, and E7 branches of the Cl1 channel system.

This water channel system leading to the Mn4 atom of the OEC has three contributing branches (E5, E6, and E7), which remain functional throughout the MD simulations. E5 has a wider cross section and the shortest distance to the OEC and lies at the D1-D2-PsbO interface (Figure 8). The entry of water begins at the junction of the PsbO-Glu114, PsbO-His228, and PsbO-His231. Further progression leads to interaction with the D2-Glu310, PsbO-Arg115, D1-Tyr73, PsbO-Arg152, D1-Glu65, D1-Asp59, D2-Glu312 and D1-Arg334 salt-bridge, D1-Asp61, D2-Lys317, and D1-Asn181, and then finally toward the Mn4 site of the OEC. As expected from the large width of this channel, DMSO is found penetrating through this channel in the 5B66 crystal structure.

The E6 tributary is situated very close to the origin of E5. Water through this channel enters the protein scaffold near the interface between the carboxylate clusters PsbO-Asp222, PsbO-Asp223, PsbO-Asp224, and PsbO-Lys188. This water channel merges with E5 in the region near D1-Glu65 and PsbO-Arg152. The channel corresponding to E7 is a subsidiary of E6 and originates at the interface between the PsbO, PsbU, and CP47. In our simulations, we observed that water inside this branch can enter from multiple directions due to the very close proximity to the bulk. Water enters the protein matrix near the interface of PsbO-Arg189 and PsbU-Asn11 and then passes through PsbO-Ala185, PsbO-Arg162, and PsbO-Thr153. The E7 channel merges with E6 near the PsbO-Asp224 and PsbO-Lys188 (Figure 8).

**Figure 9:**
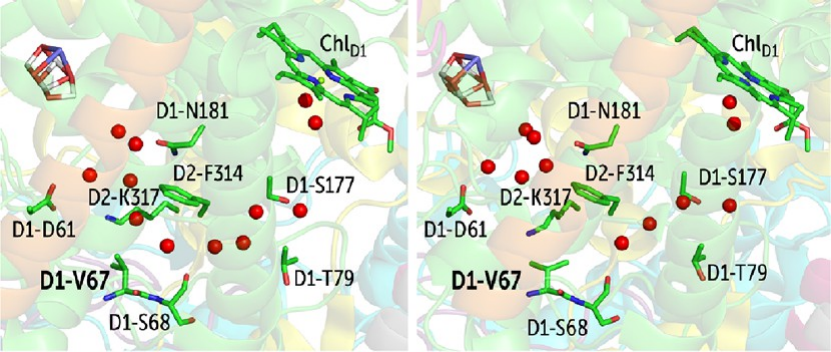
Transient water wire formation that is dependent on the conformation of D1-Val67: open (left) and closed (right) configurations determined by the side-chain rotation of D1-Val67.

We also observe a transient branch in the Cl1 channel system, which becomes active for certain time intervals of our MD simulation. Close inspection reveals that opening and closing of this channel is determined by rotation of the D1-Val67 side chain (Figure 9). The open conformation leads to filling with water toward the hydrophilic region near D1-Asp61. However, given that this transient channel has no direct connectivity to the bulk, it is not clear at the moment what role this channel might play in the regulation and function of the OEC. Interestingly, the water wire in this channel terminates near the chlorophyll Chl_D1_ of the reaction center, i.e., the pigment considered to be the primary electron donor of PSII.^75−78^

#### 2.3.3 O4 Water Channel System

**Figure 10:**
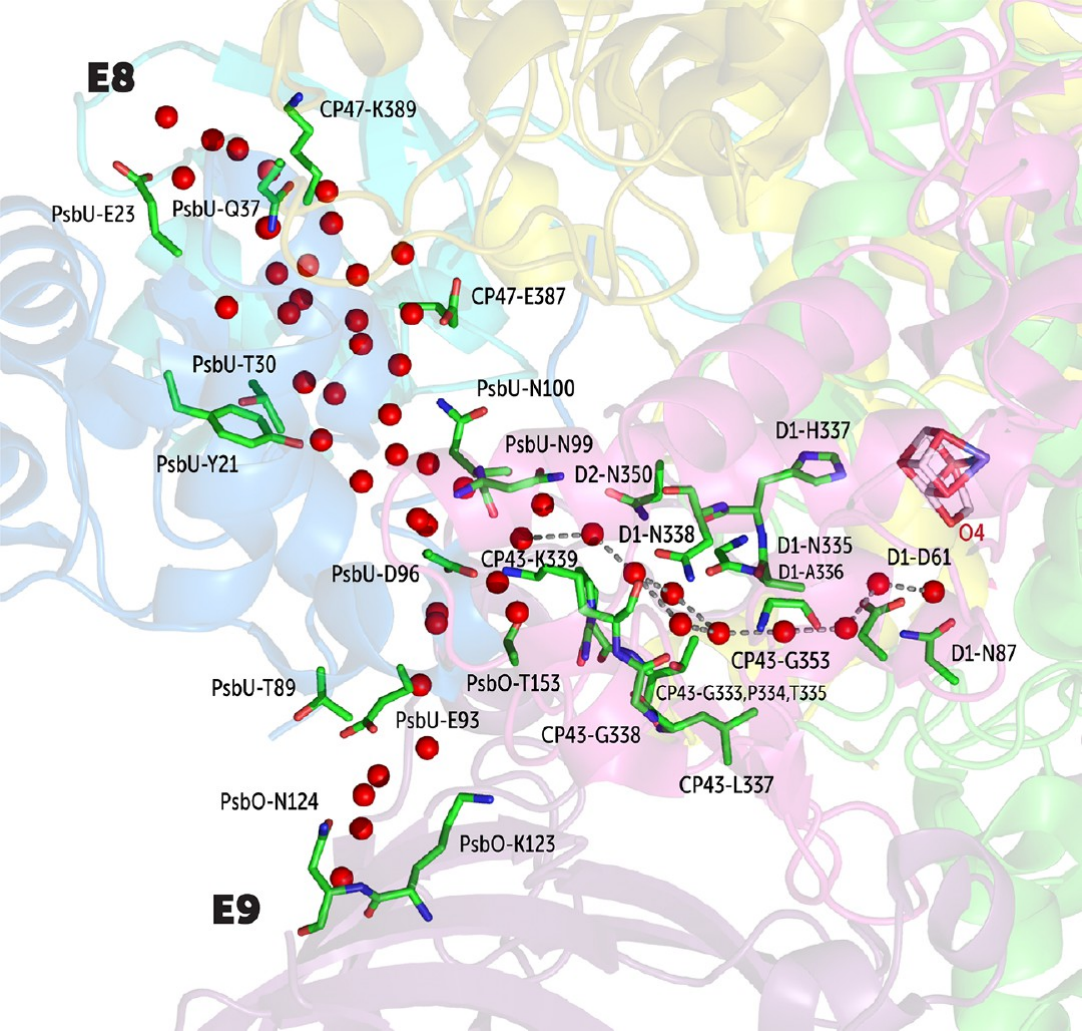
Depiction of the O4 channel system. The E8 and E9 branches merge close to the PsbU-Asp96/CP43-Lys339 pair.

The O4 system is the longest of the OEC-associated channel systems (Figure 10). In total, we observe two tributaries connecting it to the bulk, E8 and E9. Both entry points remained hydrated throughout the MD simulations. E8 is at the interface between the PsbU and CP47 proteins. The water exchanging through E8 first interacts with CP47-Lys389, PsbU-Glu23, and PsbU-Gln37. Thereafter, water passes through the region around PsbU-Tyr21, PsbU-Thr30, and CP47-Glu387 and subsequently toward the salt bridge formed by PsbU-Asp96 and CP43-Lys339. Further residues defining the channel are D2-Asn350, CP43-Pro334, D1-Asn335, and CP43-Leu337 toward the O4 atom of the OEC. The water between the PsbU-Asp96 and CP43-Lys339 salt bridge and the O4 forms a linear water wire consisting of up to 10 water molecules. DMSO solvent can be seen at the entrance of this channel (near PsbU-Tyr21) in the crystallographic model of PSII by Tanaka et al.^43^ The E9 branch is located at the PsbU–PsbO interface, beginning around a salt bridge formed by PsbU-Glu93 and PsbO-Lys123, PsbO-Asn124 and PsbU-Thr89. A DMSO molecule is at the periphery of this channel in the 5B66 model. Water molecules through both the E8 and E9 branches merge at a region near the PsbU-Asp96 and CP43-Lys339 salt bridge. It is noted that in contrast to crystallographic models,^41−43^ the MD results do not show any “break” in the continuity of waters and hence in the connectivity between the OEC and the bulk through the O4 channel. Such breaks observed in crystallographic models may therefore be artifacts of dehydration and not genuine structural features of the enzyme in its physiological state.

#### 2.3.4 Overview and General Remarks on Donor-Side Channel Systems

It is clear from the results and discussion above that all major channels leading from the bulk toward the OEC have multiple entry points. The origin of all water channels described here lies in the interface between the core polypeptides (i.e., D1, D2, CP43, and CP47) and the extrinsic proteins (PsbU, PsbV, and PsbO). The entry points for the water in the Cl1 channel system lie at the PSII dimer interface, whereas those of the O1 and O4 channel systems are away from the dimer interface, and hence PSII dimerization should not affect them. As we observe that a few of the channels originate around the conformationally flexible C-terminal residues (especially for the O1 channel system) and also involve salt-bridge interactions between core polypeptides and extrinsic proteins, the flexibility of such residues and ion-pairs can determine the opening or closing of channels or even the amount of water flux. Therefore, it is important to derive information on the channels from a dynamic treatment rather than from simple inspection of static crystallographic models, which typically have unresolved and disordered regions close to terminal residues.

Among the channels described above, we reported specific regions that contain highly ordered waters. The water molecules in these water “wires” do exchange with the bulk during our simulations, with the exception (at least within the time scale employed in this work) of the waters associated with Y_Z_ (Figure 6). The gated E4 channel, which has not been previously described in atomic detail, gains particular significance in this respect because it provides a structural basis for the possibility of proton translocation from the Y_Z_ site. Ordered waters reflect a highly organized hydrogen bonding network that is typically associated with Grotthuss-like proton transfer. This is the case for the ordered water molecules of the O4 network that are close to the OEC and that have been discussed as a possible pathway for proton release to the bulk.^23,79,80^ This is most plausible if O4 is protonated in the S_0_ state of the OEC,^79^ in which case, the proton would be released through the O4 network during the S_0_ → S_1_ transition. However, other studies favor O5 as the unique protonated bridge in the S_0_ state.^81−83^ The question of proton release is even more significant for the later stages of the catalytic cycle, the S_2_ → S_3_ transition that prepares the cluster for oxygen evolution and the S_3_ → S_0_ transition that evolves O_2_ and resets the catalyst to its lowest oxidation level. Here, we note a recent study by Noguchi and coworkers^84^ who investigated the effect of the D1-Glu65Ala mutation and suggested that the Cl1 channel serves as a single proton exit pathway in the S_3_ → S_0_ transition, whereas proton transfer in the S_2_ → S_3_ transition occurs through multiple pathways. Attribution of proton egress functionality to the Cl1 channel is in line with several other experimental and theoretical studies.^22,30,33,85−89^ On the other hand, the conclusion regarding the nonspecificity of proton release in the S_2_ → S_3_ transition^84^ mirrors the suggestion by Gunner and coworkers that all pathways are practically interconnected in the vicinity of the OEC, such that a proton released from any site of the cluster can in principle access all paths.^90^ The complexity of these events and the persisting uncertainties regarding the precise structures of intermediates, particularly in the S_3_ state of the OEC,^47,70,91−95^ pose continuing challenges for the detailed molecular understanding of proton transfer and associated channel specificity.

Conversely to the ordered water networks, high water mobility has been assumed to be beneficial for substrate delivery and in this case, the O1 channel system (which does indeed exhibit the highest rate of water exchange also in our simulations) has been associated with the delivery of water to the OEC for this reason.^33^ Our simulations confirm relative differences in water mobility among these channels. However, this information does not allow us to assign functional roles because substrate binding and OEC catalytic cycling are strictly not incorporated in our simulations. Nevertheless, we would like to note that correlations of water mobility with function (i.e., the hypothesis that low mobility is more consistent with proton transfer whereas high mobility is more consistent with substrate delivery) are not self-evident. For example, water delivery may need to be *precise* more than it needs to be *fast*. This would be in line with the paramount importance of controlling reactivity at the highly oxidizing donor side of the PSII complex^9^ whose overall chemistry is kinetically limited by the acceptor-side reactions (reduction of the terminal electron-acceptor plastoquinone Q_B_). In this sense, the O4 channel may not yet be excluded as a water-delivery system; in fact, it is favored for this role by multiple steered molecular dynamics simulations of water permeation energetics^20^ and by several studies that identify the O4 and Mn4 sites of the OEC as most accessible to small polar molecules such as ammonia and methanol.^34,35,96−104^ In conclusion, assignments of functional roles for the donor-side channels, particularly in relation to S-state progression, remain uncertain and will require further studies to be reliably established.

### 2.4 Architecture of Water Channels on the Acceptor Side of PSII

More extensive differences in hydration with respect to available crystallographic models are identified at the stromal (acceptor) side of PSII. This contains the redox-active plastoquinones, the nonmobile primary electron acceptor Q_A_, and the mobile terminal acceptor Q_B_ responsible for transporting reducing equivalents further along the photosynthetic chain to cytochrome b_6_f. A non-heme iron interfaces the Q_A_ and Q_B_ sites. The role of the iron-bound (bi)carbonate remains a subject of active research, but it is expected that bicarbonate and water are implicated in the protonation of the terminal acceptor Q_B_.^105,106^ Unlike the OEC site that is deeply buried within the protein matrix, the non-heme iron site is situated at the solvent-accessible stromal region; therefore, the hydration level in crystallographic models of this region is always and consistently lower compared to the lumenal region. It is emphasized that internal water molecules in the stromal side of the protein complex are far less likely to be conserved compared to internal water in the lumenal side that is capped by a host of extrinsic proteins.^14^

**Figure 11:**
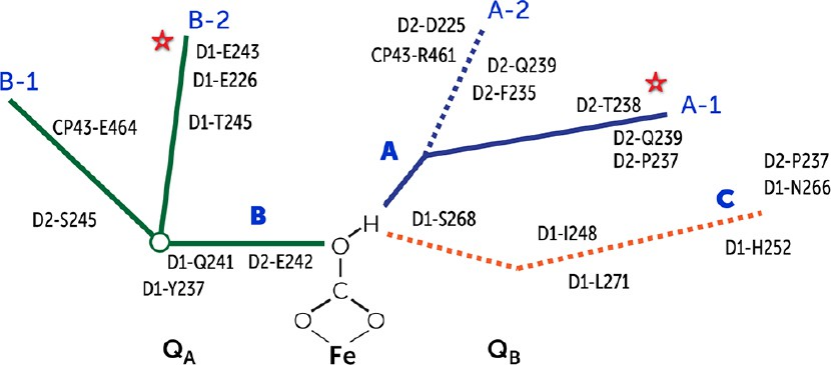
Schematic figure showing the three water channel systems connecting the non-heme iron site of PSII with the stromal bulk. Asterisks indicate positions of DMSO molecules found in the 5B66 crystallographic model. Dotted lines imply the transient nature of respective water channels due to protein conformational dynamics.

Our MD simulations lead to three crucial observations: (a) the stromal side of PSII is more hydrated than implied by even the highest-quality crystallographic models; (b) there are three well-organized water channel systems (labeled A, B, and C in the following, see Figure 11) connecting the bulk with the non-heme iron site, with multiple water entry/exit points; and (c) specific channels leading from the bulk toward the Q_B_ pocket are gated by protein conformational changes (side-chain motion and loop dynamics). Just like the channels observed around the OEC, the channels in the acceptor side differ from each other in their respective width, water influx, and local protein conformational dependence.

#### 2.4.1 Channel A

**Figure 12:**
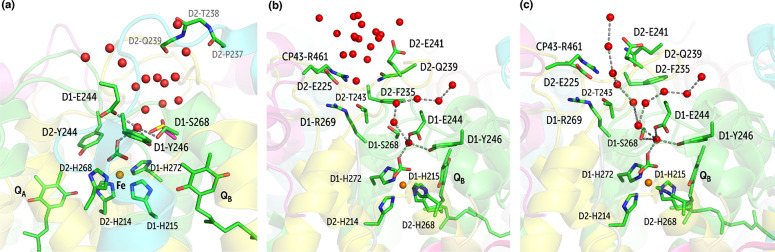
(a) Identification of the A1 water network leading from the bulk to the non-heme iron site; (b) A2 channel in the closed state, channel A1 is also depicted; (c) A2 channel in the open state, merging with channel A1.

This is the widest and shortest channel on the acceptor side. It remains hydrated throughout the simulation, and water can exchange easily between protein and bulk. The channel has two entry points for water, labeled A1 and A2 (Figures 11 and 12). In the case of A1, the water first interacts via hydrogen bonding with the backbone carbonyl of D2-Pro237, D2-Thr238, D2-Gln239, and D1-Tyr-246. Upon entry, the water establishes hydrogen bonding interaction with D1-Ser268. Aided by the large flexibility of its side-chain, D1-Ser268 “guides” the water between the bicarbonate group (HCO_3_^–^) and D1-Tyr-246. This particular region is special because at a given time, a single water can simultaneously hydrogen-bond with the D1-Ser268, bicarbonate, D1-Tyr-246, and D1-Glu244. Water molecules in the region have large residence times, which may have important implications for the redox and proton transport events at this site. Interestingly, the existence of this channel might be inferred by the 5B66 crystallographic model of Tanaka et al.^43^ (a DMSO molecule is found at the entrance of this channel, see Figure S7); however, it is dehydrated compared to the MD simulations. In the “worst-case” of the XFEL-SFX models, no water molecules are present and hence the existence of the channel is not obvious.

The A2 branch is transient in nature and depends on the local conformational dynamics of the protein. Water entering A2 interacts with the D2-Asp225 and CP43-Arg461 salt bridge, D2-Glu241, D2-Gln239, D2-Thr243, and D1-Arg269 (Figure 12). Based on our simulations, the region around D2-Gln239 acts as a gate for this channel. Owing to its high side chain mobility, it adopts open and closed conformations that allow water to merge toward the A1 branch near D1-Ser268 (Figure 12 and S8). D2-Phe235 is another important residue controlling this specific channel, since its swaying motion allows modulation of the width of the channel, regulating water passage. In the 5B66 crystal structure, this channel is dehydrated due to the closed conformation of side-chain of D2-Gln239 (Figure S7). A few waters can be seen at the entrance of this channel in the crystallographic model, resembling the hydration structure predicted by our MD simulations when the channel is closed. We will not speculate on the possible functional relevance of this channel, but we note that a site-directed mutagenesis study^107^ involving the D1-Arg269Gly substitution demonstrated impaired oxygen evolution and photosynthetic growth.

#### 2.4.2 Channel B

**Figure 13:**
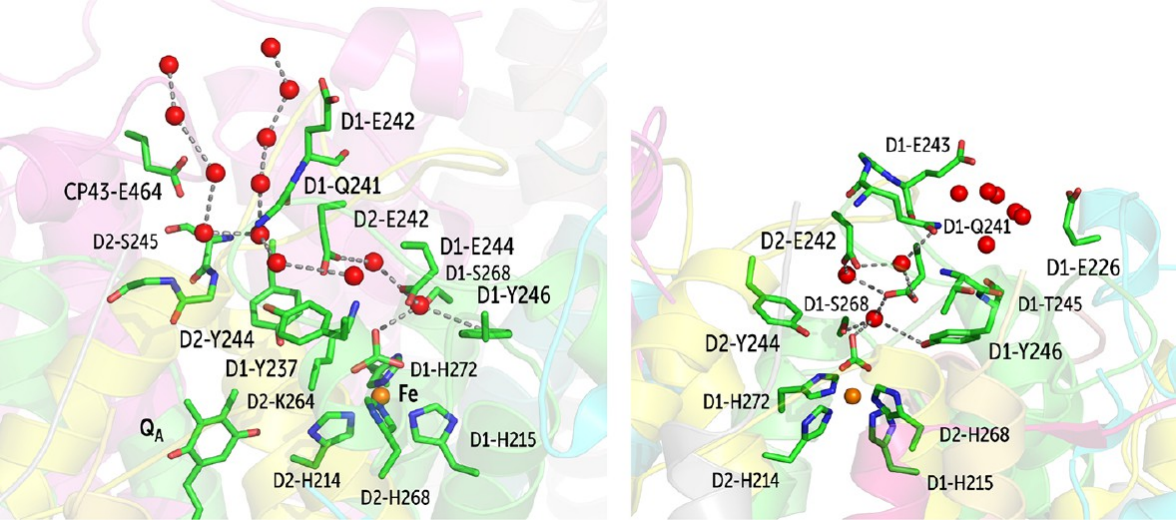
B1 (left) and B2 (right) water branches connecting the bulk with the NHI site.

This channel provides a gateway for bulk water to access the NHI site through an extensive hydrogen bond network. This water channel has two obvious entry points, labeled B1 and B2 (Figure 13). In case of B1, the water interacts with D1-Glu242 and CP43-Glu464, while a series of hydrogen bonding interactions are facilitated by D2-Ser245, the peptide -NH groups of D2-Val247 and D2-Met246, backbone carbonyls of D1-Gln241, D1-Tyr237, and the D2-Glu242/D2-Lys264/D1-Glu244 triple salt bridge (Figure 13). We observe that exchange of water from this channel to the NHI site is highly dependent on the conformational flexibility of the D2-Glu242 and D2-Lys264 salt bridge. For the majority of the simulation time, water exchange does not occur. However, the connectivity of the channel to the NHI site through hydrogen bonding remains intact. This channel can also be seen in several high-resolution crystallographic models of PSII.^33,41,43^

Branch B2 is slightly shorter in length compared to B1. Water entering through B2 first interacts with D1-Glu243 and D1-Glu236 and thereafter establishes hydrogen bonding with the side-chains of D1-Gln241 and D1-Thr245. The side-chain of the D1-Gln241 is further hydrogen bonded to the water molecules of the B1 branch. B2 remains hydrated throughout our MD simulations. This particular channel can also be seen in crystal structures by Tanaka et al.^43^ and Umena et al.^41^ (Figure S9). A DMSO molecule can also be seen at the entrance of this channel in the 5B66 model.

#### 2.4.3 Channel C and the Q_B_ Pocket

**Figure 14:**
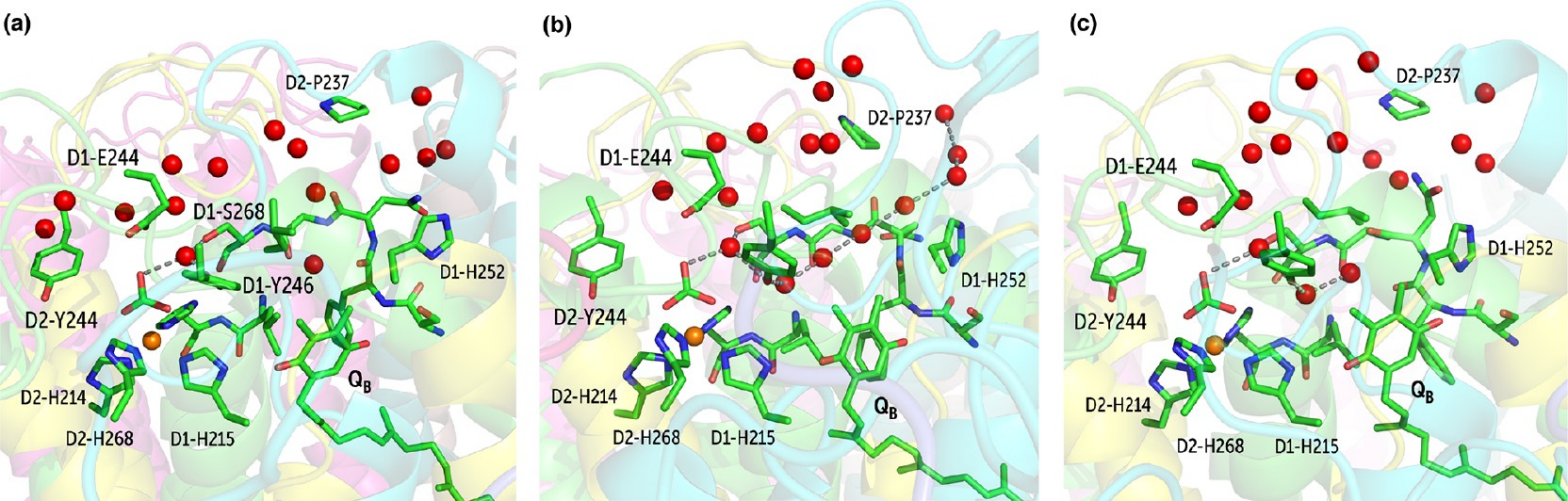
(a) Entry of water into channel C blocked by the hydrophobic side chains of D1-Ile248 and D1-Leu271; (b) formation of water wire connecting the bicarbonate site through the Q_B_ pocket with the bulk; (c) Q_B_ pocket water trapped because exit is restricted by the side chain conformation of D1-Asn266.

This channel is unique and has not been previously resolved crystallographically. Water present in this channel interacts with the NHI site through the Q_B_ hydrophobic pocket, and hence, it is expected to play a crucial role in Q_B_ protonation. Water inside this channel can exchange with the bulk from two sides: (a) through channel A and (b) through an area between D1-Ile248, D1-Asn266, D2-Pro237, and D2-Phe235 (Figure 14).

The entry of water through both endpoints is gated by the local conformation dynamics of the protein. Specifically, the entry of water to channel C through channel A depends on the side chain rotations of D1-Leu271 and D1-Ile248: the cavity is open when the side-chains are facing away from each other; otherwise, it is closed (Figure 14). We observe that the gating by these residues in the Q_B_ pocket is the strongest determinant for the functionality of this channel. The Q_B_ pocket can only hold a maximum of two water molecules at a time.

With the knowledge about the existence of this channel derived from our MD simulations, we revisited the crystallographic models and identified a structural “trace” of this previously unknown channel in the crystallographic model of Umena et al.^41^ (but not in the model of Tanaka et al.^43^) where D1-Ile248 is disordered and present in two conformations, which correspond to the open and closed conformations of our computational model with occupancies of 70 and 30%, respectively (Figure S10).

A crucial point concerns the hydration of the Q_B_ pocket. We observed that the presence of two water molecules inside the Q_B_ cavity was initially predicted by our 3D-RISM calculations. Subsequent MC/MD calculations never removed these waters from the cavity. To further confirm that the hydration of this cavity is not an artifact of the initial system preparation, we performed MD simulations after *removing* these waters. We observed that the cavity is filled up again with water within the first 5 ns of the MD simulation, confirming that the presence of these two water molecules represents a physiological hydration state of the system.

The entry and exit of the water through D1-Ile248, D1-Asn266, D2-Pro237, and D2-Phe235 regions depend on the motion of two loops, the first one consisting of D1-Asn267, D1-Asn266, D1-Phe265, and D1-Ser264 and the second one consisting of D2-Gln239, D2-Thr238, D2-Pro237, D2-Asn236, and D2-Phe235. These two loops were found to be highly flexible in our MD simulations (Figure S11). In addition, the side chain of D1-Asn266 was also found to play a crucial role in the opening and closing of this channel from this particular end-point. This implies that even if water enters through channels A and B into the Q_B_ pocket, it may not leave until the other end is open. This also leads to large residence times of the waters in the Q_B_ pocket. Interestingly, the exit of the waters through these loops is at the very origin of the A1 branch. It is important to highlight the special role of the loop consisting of D2-Gln239, D2-Thr238, D2-Pro237, D2-Asn236, and D2-Phe235, since it forms the gateway for A1 and subsequently controls movement of water through C and A2. Although this particular loop region was found to be in the open state in many crystal structures, no water molecules were resolved. D1-His252 is present at the entrance of this channel; this residue is suggested to be critical^108^ in providing the first proton to the Q_B_ through D1-Ser264.^109^

#### 2.4.4 Hydrogen Bonding Network Around the NHI Site

The hydrogen bonding network between the protein residues and channel waters is dynamic yet retains a high and clearly identifiable structure and organization. As this region is close to the protein–bulk interface, it is subject to high perturbation from the bulk. It is also evident that the side chains of many residues around the NHI are highly flexible compared to more deeply buried residues. Perhaps counterintuitively, side-chain flexibility is found to be crucial for *maintaining* organization in water channels leading to the NHI site because by easy rotation of, for example, D1-Ser268, the residue can always either establish a hydrogen bonding interaction with the incoming water or stabilize (and hence maintain the structure of) the hydrogen bonded water tetramer near the bicarbonate. We note that recently targeted mutagenesis by Forsman and Eaton-Rye^110^ highlighted the importance of D1-Ser268 in conjunction with the bicarbonate for proton transfer events in the formation of Q_B_H_2_. The importance of D1-Ser268 is also highlighted by the site-directed mutagenesis studies of Alfonso et al.,^111^ who observed that serine to proline substitution leads to a decrease in the electron transfer rate between Q_A_ and Q_B_ and stabilization of S_2_Q_B_^–^ and S_3_Q_B_^–^ states.

The triple salt bridge consisting of D1-Glu244, D2-Lys264, and D2-Glu242 is significant in terms of directly interacting with the incoming waters from channels A and B and stabilizing them through extensive hydrogen bonding. This structural observation derived from the MD simulations may relate to experimental results of site-directed mutagenesis^112^ on D1-Glu244 and D2-Tyr246, which resulted in impaired oxygen evolution, implying that these residues maintain an important hydrogen bonding network critical for the smooth function of the acceptor side. Similarly, recent site-directed mutagenesis study by Khaing et al.^113^ on D2-Tyr244 showed impaired electron transfer between Q_A_ and Q_B_, including disruption in the PSII assembly and in the back-reaction with the S_2_ state of the OEC. In addition, Fourier transform infrared spectroscopy studies by Hienerwadel and Berthomieu^114^ documented a hydrogen bond network from the non-heme iron toward the Q_B_ pocket. Based on our MD results, we do see such a strong network, which involves the non-heme iron, D1-Glu244, D1-Ser268, D1-Tyr246, and two water molecules in the Q_B_ pocket. Similarly, Takahashi et al.^115^ claimed that one of the two tyrosines participates in a direct hydrogen bonding interaction with the bicarbonate ligand. Based on our simulations, D2-Tyr244 is serving as hydrogen bond donor to both the bicarbonate and the nearby D2-Met246.

#### 2.4.5 Role of Hydration in Q_B_ Protonation in PSII and the Bacterial Reaction Center

**Figure 15:**
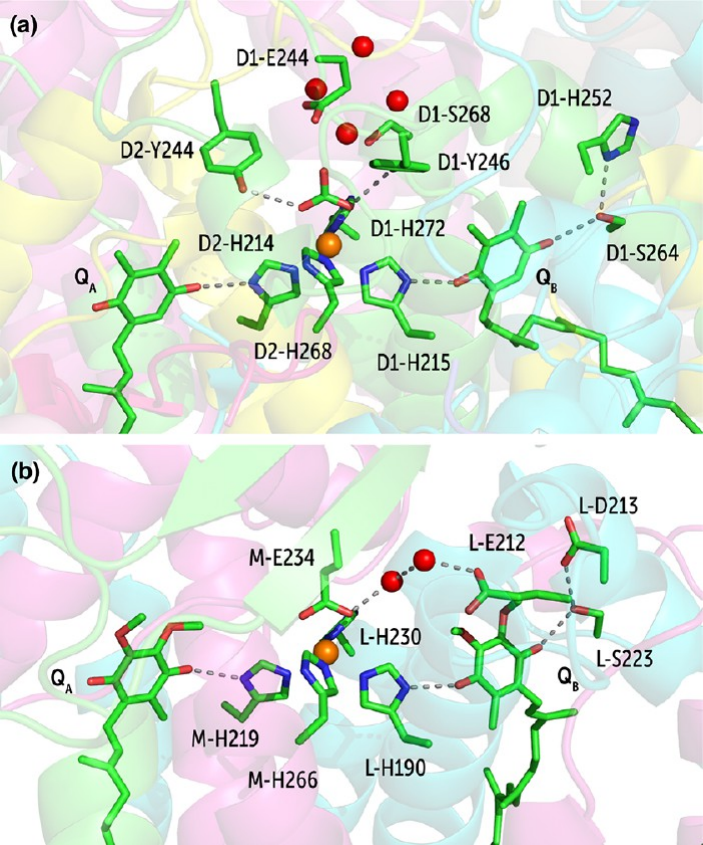
Differences in the protein environment around the analogous quinones in (a) the Photosystem II (PDB ID 3WU2) and (b) the bacterial reaction center (PDB ID 3I4D).

PSII and the bacterial reaction center (bRC) share striking similarity in the architecture around the quinones and non-heme iron site but with some key important structural differences (Figure 15).^116^ This is clearly reflected in the electron transfer kinetics, for example, the rates of first and second electron transfers from Q_A_ to Q_B_ are different in bRC and PSII. The first electron is transferred within 100 μs in bRC,^117^ whereas it takes 400 μs in the case of the PSII.^118^ The second electron transfer event is much slower, at 1 ms in bRC^117^ and 0.6–0.8 ms in PSII.^118^ In the reaction center of purple bacteria, after the first electron transfer from the primary acceptor ubiquinone Q_A_ to the terminal acceptor ubiquinone Q_B_, the first proton is known to be delivered through L-Ser223, which in return receives a proton from L-Asp213 (Figure 15). After re-reduction of Q_B_ by Q_A_, the second proton may be provided by the L-Glu212 residue.^119,120^ Sugo et al.^121^ suggest that the second proton is derived from L-His190 instead, whereas L-Glu212 is involved in water-mediated proton transfer to the deprotonated L-His190. Assuming that PSII operates in a similar fashion, it is speculated that the first proton delivered to Q_B_ is derived from D1-Ser264, which in return receives the proton from D1-His252 that has access to the bulk. Some experimental and theoretical studies suggest instead that the second proton is derived from D1-His215.^105,106,116^

The large time-scales of the sequential electron and proton transfer processes suggest strong coupling between the conformational changes of the protein and redox events. Water can be functionally important given the proximity of the non-heme iron site to the bulk in both the PSII and the bRC. Interestingly, the role of overall protein hydration in the Q_A_ to Q_B_ electron transfer kinetics has been discussed in the case of the bRC.^56,57^ Again, it highlights the significance of water in providing functional flexibility to the protein scaffold and established its mechanistic role in redox events around the Q_B_ site. Similarly, the role of hydration in terms of the electron transfer kinetics in the acceptor side has been demonstrated also for PSII.^60,61^ Dehydration leads to impaired electron transfer from the Q_A_ to Q_B_ or no electron transfer at all. Therefore, correctly and fully hydrated PSII models are the key for understanding the system’s overall structure and function.

The Q_B_ pocket waters discussed above in connection to channel C have not been previously identified in existing crystal structures of PSII. Given their proximity to the bicarbonate and Q_B_, and in view of the connectivity to the bulk, they may be involved in proton transfer within the Q_B_ cavity. Based on the MD simulations and previous results from various experimental and computational studies, the Q_B_ pocket waters can play two roles: (a) mediate proton transfer from the bicarbonate or bulk to Q_B_, and (b) mediate proton transfer to the deprotonated D1-His215. In addition to the role of the water channels in proton transfer, a recent work by Fantuzzi et al.^122^ demonstrated the role of the water channels in dioxygen diffusion toward the non-heme site. Assuming that D1-His215 transfers the second proton to Q_B_ and becomes doubly deprotonated, the Q_B_ pocket water molecules can transfer a proton to D1-His215. In this case, the proton can enter from the bulk or from the bicarbonate itself. Ishikita and co-workers^109^ postulated the presence of the Q_B_ pocket water for the protonation of the D1-His215 anion.^123^ In addition, the second proton transfer can also be initiated by the Q_B_ pocket waters due to their close proximity; however, this would require some degree of rotation of the Q_B_ head-group. This particular proposal remains to be investigated, and it would be interesting to see how the electron transfer events are coupled to the conformational changes in the protein around the Q_B_ region.

## 3 Conclusions

In this work, we presented large scale molecular dynamics simulations of membrane-bound Photosystem II to understand its hydration structure, employing a coherent protocol for the generation of a fully hydrated system before production level simulations. Three aspects were targeted: (a) overall hydration of the PSII and comparison with the recent and previously known crystallographic models, (b) water channel architecture around the OEC, and (c) water channel architecture at the acceptor side of PSII.

The hydration count predicted by our MD simulations best matches with the structure reported by Tanaka et al.,^43^ which represents the highest-resolution synchrotron-based crystallographic model of PSII. Other crystallographic models display lower water counts. At the extreme, recent models derived from XFEL crystallography were found to be severely dehydrated, not only in the periphery of PSII but also internally and even around the OEC.

Our results map in great detail the three water channel systems connecting the OEC with the bulk. We document the multiple water entry/exit points of these channels and identify a number of previously unknown transiently formed water pathways. Protein conformational dynamics plays a key role in the functionality of several channels. Especially, we highlight the role of PsbV-Tyr137 in conformationally gating the water channels and its possible involvement in proton egress from the Y_Z_ network. Almost all water channels are interfaced between the core proteins and extrinsic proteins, underlining the role of extrinsic protein in regulating water access to the OEC and proton egress to the lumen.

On the acceptor side of PSII, we identify an extensive and well-organized water channel system, which typically remains dehydrated in most experimental structures. An important discovery is a previously unknown channel that is responsible for hydration of the Q_B_ pocket and hence may play an important role in proton translocation around the non-heme iron site or even directly in the protonation of the terminal acceptor Q_B_.

The present study offers new insight into the equilibrium water content of PSII and the dynamic nature of its static and transient water channels. The fully hydrated structures of PSII, provided as an open-data collection of supplementary PDB files, can be used for evaluating the hydration content of crystallographic models and serve as starting point for future investigations into the precise mechanistic role of water in donor- and acceptor-side processes.

## Supporting Information Available

Detailed description of methodology including system setup, parameterization, and protocols used for the molecular dynamics simulations; analysis of molecular dynamics trajectories (Figures S1–S3); distribution of 3D-RISM positioned water in the Q_B_ cavity (Figure S4); and comparisons with crystallographic models and depiction of conformational flexibility of key residues (Figures S5–S11) (PDF)

## Notes

Original PDB files from the MD simulations selected to depict representative hydration states of the water channels identified in the present work are provided as an open-access data set hosted by the Open Research Data Repository of the Max Planck Society at https://doi.org/10.17617/3.B2AKNU.
