# Supplementary material for: Functional Water Networks in Fully Hydrated Photosystem II
Source: J Am Chem Soc. 2022 Nov 22;144(48):22035–50. doi: 10.1021/jacs.2c09121 (PMC9732884; doi:10.1021/jacs.2c09121)
Supplement: Supplementary file 1 — ja2c09121_si_001.pdf [file ja2c09121_si_001.pdf]

Functional Water Networks in Fully Hydrated Photosystem II

Abhishek Sirohiwal and Dimitrios A. Pantazis*

Max-Planck-Institut für Kohlenforschung, Kaiser-Wilhelm-Platz 1, 45470 Mülheim an der Ruhr, Germany

*E-mail: dimitrios.pantazis@kofo.mpg.de

SUPPORTING INFORMATION

1. Methodology

1.1. System Preparation

The lipid-bilayer embedded model of Photosystem II is based on the high-resolution dimeric crystal structure (1.9 Å) of *Thermosynechococcus vulcanus* (PDB ID: 3WU2).[1] In the current setup we choose one of the monomers to build the entire system and first aligned it in a way such that the *z*-axis is normal to the membrane place. This is achieved using the OPM (Orientation of Protein in Membranes) web server.[2] All structurally resolved detergents used for the crystallization were removed. All the missing terminal peptide chains were completed and modelled using the *Modeller 9.21* suite[3-5] and the respective best models were selected based on the scoring functions of obtained various models. The missing atoms of amino acids were completed using the *pdb4amber* module of *AmberTools19*,[6-8] and the protonation state of the residues and the co-factors were computed using the *reduce* module.[9] The missing or unassigned ligands or co-factors were inserted using the information from other cyanobacterial crystal structures, i.e. PDB ID 2AXT[10] and 4V62.[11] Some crystallographically resolved cofactors were found to have missing atoms, which were completed using the *pymol* suite[12] and atoms were re-numbered accordingly. All crystal waters associated with the monomer were retained during the set-up and the positions of additional waters was predicted using 3D-RISM (Three-Dimensional Reference Interaction Site Model)[13-18] calculations available in *AmberTools19* (using *rism3d.snglpnt*). We employed the SPC water model[19] during the 3D-RISM calculations. Previous studies[15-16,20-23] have shown the capabilities of the 3D-RISM technique in predicting deeply buried water solvation site in bio-molecular systems. Results from the 3D-RISM calculations were further utilized to predict the explicit positions of the waters molecules using the *placevent* module.[20] A total of 5404 waters were added inside and around the protein. The complete protein-cavity water complex is placed inside a POPC (*1-palmitoyl-2-oleoyl-sn-glycero-3-phosphocholine*) bilayer of dimension 176 x 176 x 160 $Å^3$ using the *Packmol-Memgen* module[24-25] available with *AmberTools19*. A total of 397 and 393 lipids were added in the upper and lower leaflet, respectively. In addition, the box is filled up with water using *Packmol-Memgen*, up to a distance of 17.5 Å above and below the protein, resulting in a total of 111,066 water molecules in the simulation box. A distance tolerance of 2 Å is used when placing the membrane and water to avoid clashes during the minimization and equilibration process. We added 304 Na^+ and 269 Cl^- atoms to neutralize the complete system and maintain a salt concentration (NaCl) of 0.15 M in order to mimic the physiological conditions. The entire system consists of 512,341 atoms.

1.2. Parameters for Protein, cofactors, lipid bilayer and water

The electrostatic charges for the cofactors and metal sites were computed based on the MK-RESP (Merz-Kollman Restrained Electrostatic Potential) methodology.[26-27] For the organic cofactors, first the hydrogens atoms were optimized at the B3LYP/def2-SVP level[28-29] and then single-point calculations were performed at the HF/6-31G* level of theory[26,30-31] using the ORCA program[32] and RESP fitting of the charges was performed using the Multiwfn code.[33] A bonded model is employed for the computation of the RESP charges on the OEC (Mn_4CaO_5 – Oxygen Evolving Complex) and NHI (non-heme iron) sites. As a first step, a small cluster model is built around the metal sites including the side chains of the residues which directly coordinate the metal site.

The OEC is modeled in its S_1 state of the Kok-Joliot cycle, i.e. the oxidation states are Mn1(III)–Mn2(IV)–Mn3(IV)–Mn4(III) and involved ligands are Asp170, Glu354, Ala344, Asp342, Glu189, His332, Glu333, and four H_2O molecules. Similarly, the NHI site is modelled as Fe(II) with the ligands HCO_3^-, His214, His268, His215, and His272. For RESP fitting, the charges were only computed for Fe(II) and HCO_3^- as a single unit, due to underestimation of Fe(II) charges when the complete cluster is considered. The remaining ligating histidine residues were given the corresponding charges from the AMBER force-field library. These models were first optimized at B3LYP/def2-TZVP and then RESP fitting is performed at B3LYP/6-31G* level. More importantly, we restrained the charge of the backbone atoms of the residues according to the original AMBER force field[31] as such a procedure is known to produce better back-bone dynamics during the simulation.[34] The RESP charges of the chlorophylls and the heme iron site were calculated in a similar fashion. The chlorophylls and the heme-iron are ligated axially to amino acids and water molecules, wherever applicable. For example, P_{D1} and P_{D2} of the reaction center are axially ligated to histidine residues and Chl_{D1} and Chl_{D2} are axially ligated to a single water molecule. Similarly, both heme sites are bound axially with two histidine residues.

The standard protein residues were described using the *Amber14SB* force-field[35] and water is modelled using the TIP3P model.[36] We used GAFF2 (General Amber Force Field)[37] for the organic cofactors, where the appropriate atom types were automatically generated with the ANTECHAMBER module of the *AmberTools19*. We employed the LIPID17 force field[38-39] for the POPC bilayer. Earlier experimental investigations[40] found phosphatidylcholine group based bilayer active in the oxygen evolution. Several previous molecular dynamics simulation have also successfully followed the same approach of using the POPC lipid bilayer.[41-42] The parameters for the chlorophyll *a* and heme iron site were obtained directly from the literature.[43-44] Customized bonded parameters were used for the modeling of the OEC and NHI site, for example the bonds within the OEC were assigned a value of 150 kcal mol^{-1} $Å^{-2}$, whereas the bonds between the OEC and coordinating residues were assigned a value of 70 kcal mol^{-1} $Å^{-2}$. Similarly, the angles within the OEC were restrained with a value of 120 kcal mol^{-1} rad^{-2} and angles between the OEC and coordinating residues with a value of 70 kcal mol^{-1} rad^{-2}. In addition, parameters previously reported by Ishikita and co-workers[42] for the NHI site were imported in our model. The non-bonded parameters for the metals were based on their oxidation state using the Ion-Oxygen Distance (IOD) dataset available for the TIP3P model.[34,45-46] For Na^+ and Cl^-, we used the Joung–Cheatham parameters[47-48] compatible with the TIP3P model.

1.3. Molecular Dynamics Simulation Methodology

1.3.1. Minimization

The system was minimized systematically and thoroughly in order to remove energetically unfavourable clashes inside the system. All the minimization procedure is performed on the CPU version of the *pmemd* engine.

Step 1. All hydrogen atoms were optimized for a total of 1500 steps, involving 50% each of steepest descent and conjugate gradient with a restraint weight of 50 kcal mol^{-1} $Å^{-2}$ on all the heavy atoms (i.e. non-hydrogen atoms).

Step 2. The waters, ions and membrane were optimized for 20000 steps involving 50% each of steepest descent and conjugate gradient with a force constant of 50 kcal mol^{-1} $Å^{-2}$ on the protein and the cofactors.

Step 3. The complete system is minimized (15000 steps) keeping the $C_α$ atom of amino acids restrained with a force constant of 20 kcal mol^{-1} $Å^{-2}$. Positional restraints used the final minimization step were maintained throughout the equilibration process.

1.3.2. Equilibration and Production Runs

Step 1. The system was slowly heated by increasing from 10 K to 100 K within 5 ps in the *NVT* ensemble, using the final configuration obtained from the minimization procedure, while maintaining the positional restraints (20 kcal mol^{-1} $Å^{-2}$) on the $C_α$ atom of amino acids. We employed a slightly larger value of the collision frequency (5 ps^{-1}) in the Langevin thermostat. Particle Mesh Ewald (PME)[49] approach is used to treat all electrostatic interactions with a 10 Å cut-off.

Step 2. The temperature of the system is further increased from 100 K to the target temperature (303 K) within 125 ps in the *NPT* ensemble with a collision frequency of 5 ps^{-1} of the Langevin thermostat.[50]

Step 3. In the next step, we decreased the restraints on the $C_α$ atom in a step-wise manner, i.e. decreasing from 20 to 2 kcal mol^{-1} $Å^{-2}$ with an interval of 2 kcal mol^{-1} $Å^{-2}$. We ran 400 ps of *NPT* simulations for each interval, which totals to 4 ns for the complete procedure. Thereafter, all the restraints were completely removed and the system was simulated for another 2 ns.

Step 4. In the next step, we invoked the **MC/MD** module[51] of Amber18 with water as a solvent to be exchanged between the bulk and protein interior. The main objectives for using this elegant and expensive technique are: (a) to ensure that the interiors of the protein are properly hydrated during the dynamic evolution of the protein propagated by the standard MD simulation step, and (b) to dehydrate internal cavities in case 3D-RISM over-hydrated the structure. Steric grid size was carefully chosen in order to ensure that entire internal cavities of the protein are covered within. The number of Monte-Carlo (MC) move attempts in each MC cycle was set at 1,000,000, whereas the number of MD steps in each MC cycle was set at 1,000. During all of these computations, we restrained the $C_α$ atoms of the protein to 5 kcal mol^{-1} $Å^{-2}$. After a certain amount of MC cycles, we observed no further "interesting moves" that hydrated or de-hydrated the internal cavities, and thereafter we called off the MC/MD computations. The "hydration-equilibrated" PSII structure obtained after this procedure is an ideal starting point for further simulations.

Step 5. We further equilibrated the entire system for another 61 ns to ensure the POPC bilayer is properly equilibrated. The electron density profile of the POPC lipid bilayer is reported in Figure S1.

Step 6. We initiated unbiased production run for a total of 200 ns in the *NPT* ensemble. Frames were saved every 2 ps throughout the production simulations. The stability of the protein is depicted in Figure S2.

During the system heating procedure (Step 1 and Step 2), the temperature is controlled using Langevin Dynamics with a collision frequency of 5 ps^{-1}, after stabilising the temperature we switched to 1 ps^{-1} of collision frequency. In all cases, the pressure is regulated anisotropically using the Berendsen Barostat[52] with a pressure relaxation time of 2 ps and maintained at 1 bar. Bonds involving the hydrogen atoms were constrained using the SHAKE algorithm,[53] which allowed us to use an integration time step of 2 fs. Particle Mesh Ewald (PME) approach is used to treat all electrostatic interactions with a 10 Å cut-off. The equilibration and production runs were performed on the CUDA (Compute Unified Device Architecture) version of *pmemd* engine.[54-56] Equilibration and production runs were performed using the Tesla V100 and Quadro RTX 5000 graphics cards. Analysis of the trajectories of the production simulation were performed using both CPU and the GPU version of the CPPTRAJ.[57-58] For visualization, analysis and image rendering we employed PyMOL[12] and VMD.[59]

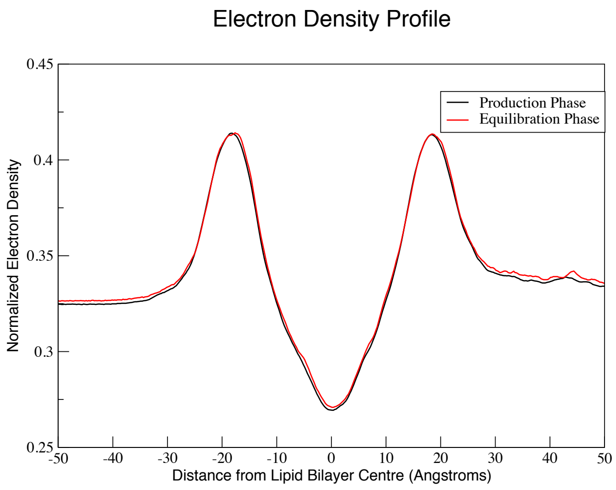

Figure S1. Electron density profile of the POPC lipid bilayer during the equilibration and production simulations. This profile was generated using the *density* option in the CPPTRAJ module.

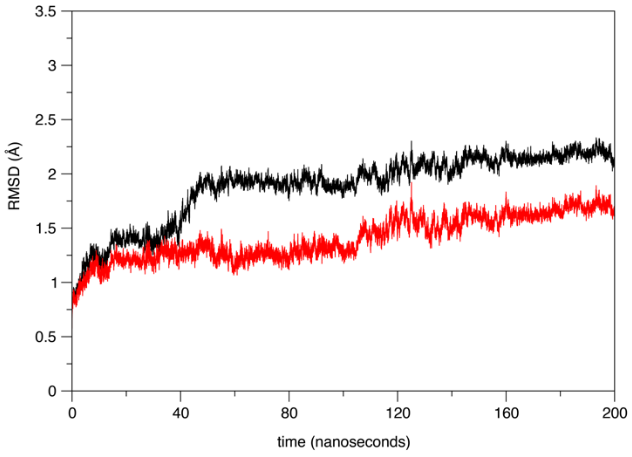

Figure S2. Structural evolution of the C_α atoms of the protein during the production simulations. Two data sets were created, i.e. complete protein evolution (red trace) and without the intrinsically disordered part (black trace).

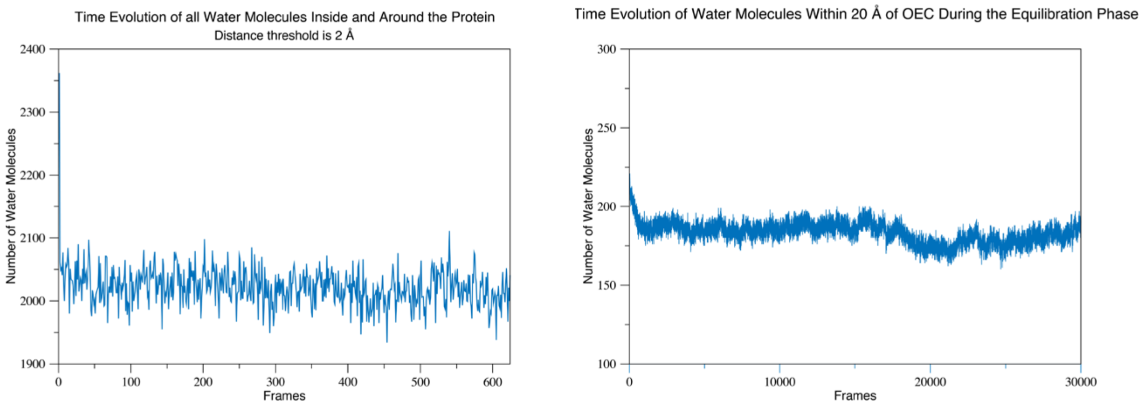

Figure S3. Evolution of the water content around the protein (left) and the oxygen evolving complex (right) during the long equilibration phase (~65 ns). The water count is performed using the *watershell* command of the CUDA enabled CPPTRAJ module of AmberTools19. We used a distance criterion of 2 Å in counting the number of waters around the protein. The number of waters were counted every ~105 ps (due to extremely high computational cost, even on the GPU) in case of the evolution of the water around the complete protein.

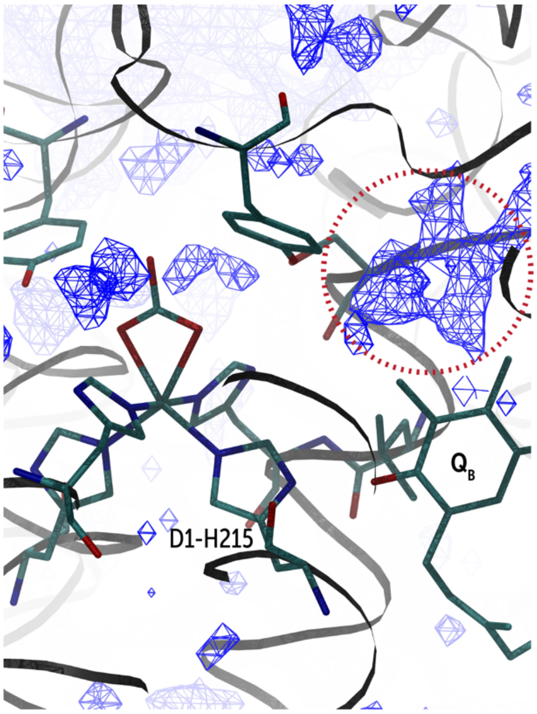

Figure S4. Distribution of the oxygen atom of the water around the Q_B pocket obtained using the 3D-RISM calculations. The circled spatial region represents the density of the Q_B pocket waters. All the hydrogen atoms are omitted for the visual clarity.

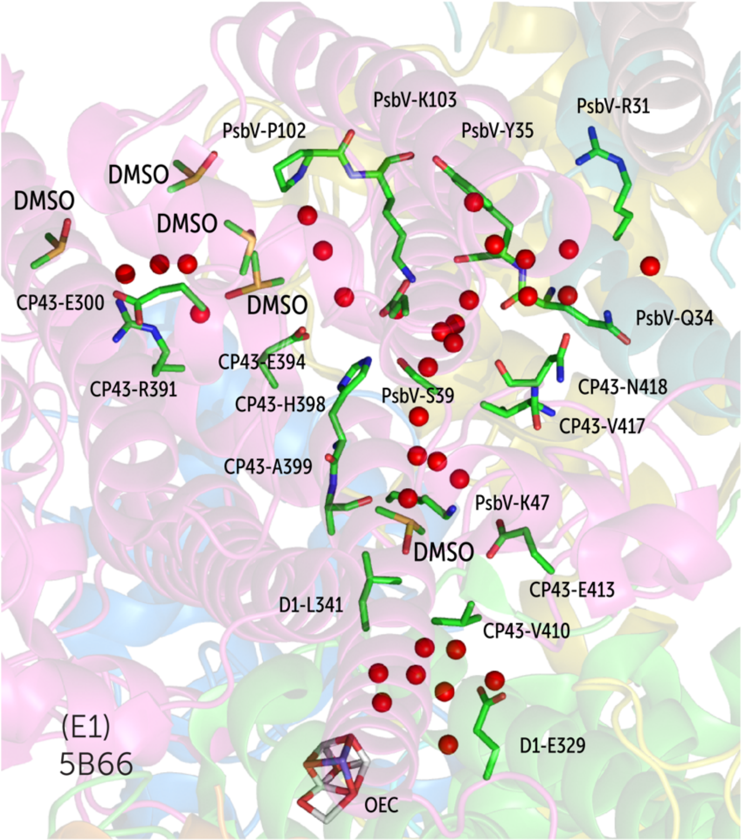

Figure S5. Depiction of the E1 branch of the O1 channel system in the 5B66 crystallographic model of Tanaka et al. DMSO molecules are indicated.

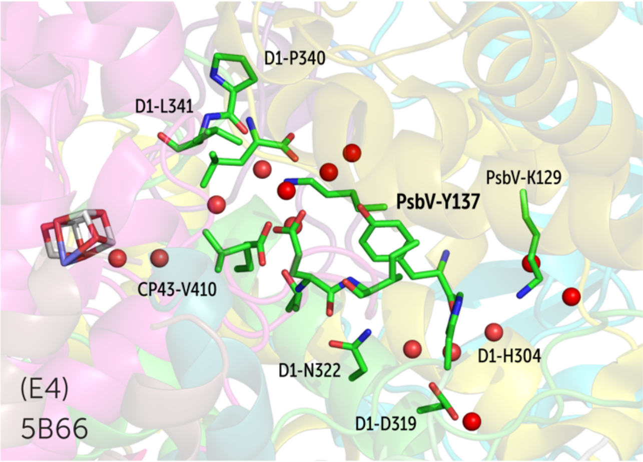

Figure S6. Depiction of the E4 branch of the O1 channel system in the 5B66 crystallographic model.

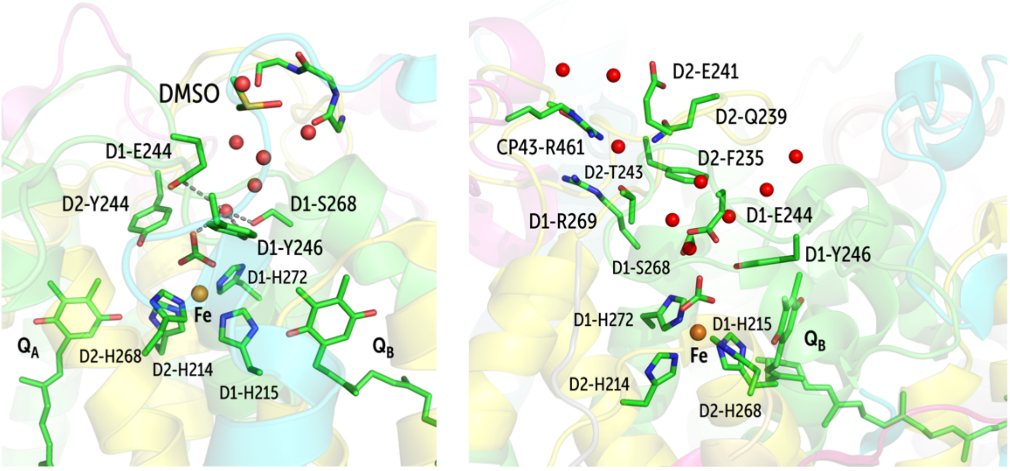

Figure S7. Depiction of the A1 (left) and A2 (right, in closed state) acceptor-side water branches in the 5B66 crystallographic model.

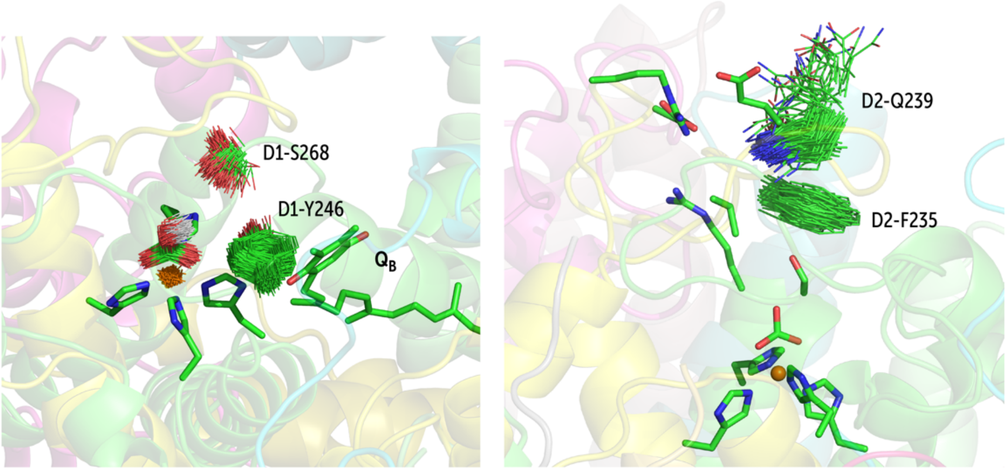

Figure S8. Conformational flexibility of key residues participating in the extensive hydrogen bonding network around the non-heme iron (left) and modulating channel A2 in particular (right).

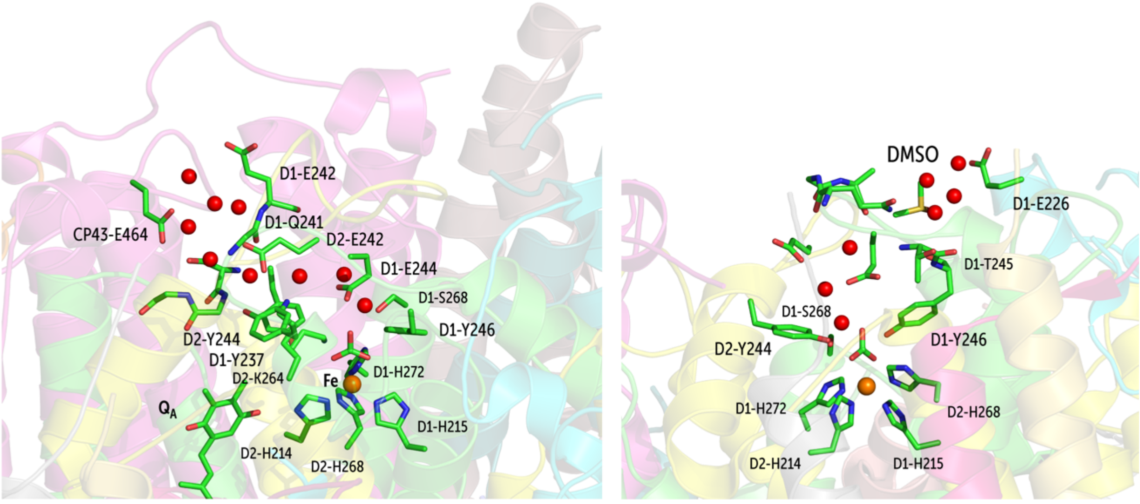

Figure S9. Depiction of the B1 (left) and B2 (right) branches in the 5B66 crystallographic model.

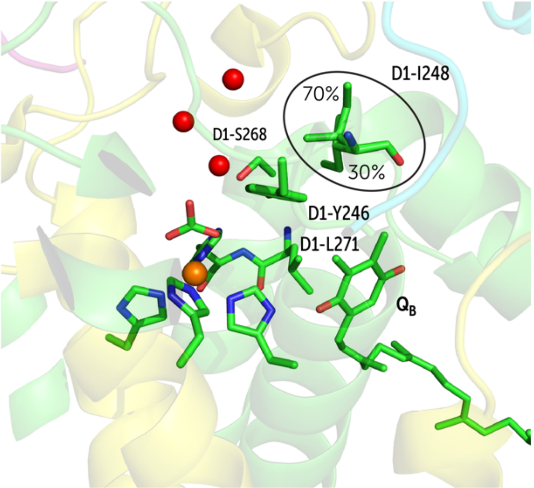

Figure S10. Disordered D1-Ile248 as observed in the 3WU2 crystallographic model of Umena et al.

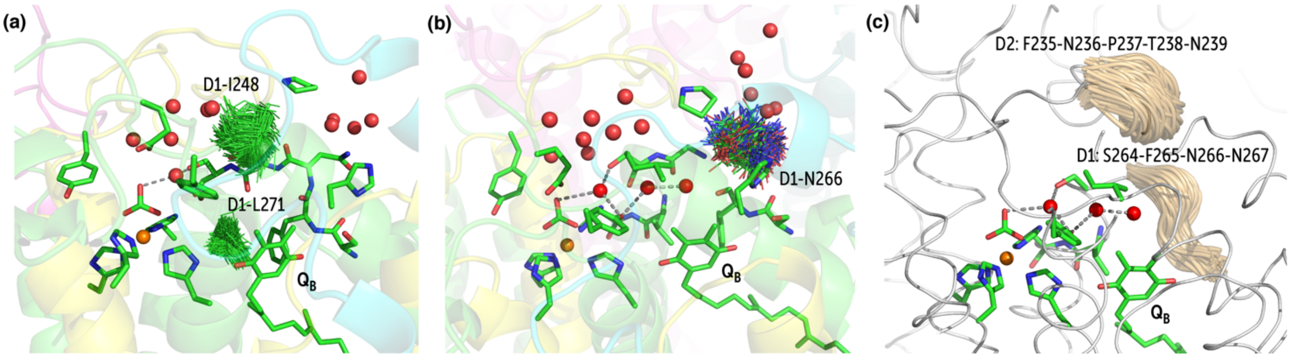

Figure S11. Depiction of conformational flexibility of: (a) the D1-Ile248 and D1-Leu271 residues; (b) the D1-Asn266 residue, and (c) two specific loop segments from the D1 and D2 protein chains, all of which play a role in controlling the hydration of channel C and of the Q_B cavity.

References

(1) Umena, Y.; Kawakami, K.; Shen, J.-R.; Kamiya, N. Crystal Structure of the Oxygen-Evolving Photosystem II at a Resolution of 1.9 Å. *Nature* **2011**, *473*, 55-60.

(2) Lomize, M. A.; Pogozheva, I. D.; Joo, H.; Mosberg, H. I.; Lomize, A. L. OPM database and PPM web server: resources for positioning of proteins in membranes. *Nucleic Acids Res.* **2012**, *40*, D370-6.

(3) Fiser, A.; Do, R. K.; Sali, A. Modeling of loops in protein structures. *Protein Sci.* **2000**, *9*, 1753-73.

(4) Sali, A. Comparative protein modeling by satisfaction of spatial restraints. *Molecular Medicine Today* **1995**, *1*, 270-277.

(5) Martí-Renom, M. A.; Stuart, A. C.; Fiser, A.; Sánchez, R.; and, F. M.; Šali, A. Comparative Protein Structure Modeling of Genes and Genomes. *Annu. Rev. Biophys. Biomol. Struct.* **2000**, *29*, 291-325.

(6) D.A. Case, I. Y. B.-S., S.R. Brozell, D.S. Cerutti, T.E. Cheatham, III, V.W.D. Cruzeiro, T.A. Darden, R.E. Duke, D. Ghoreishi, G. Giambasu, T. Giese, M.K. Gilson, H. Gohlke, A.W. Goetz, D. Greene, R Harris, N. Homeyer, Y. Huang, S. Izadi, A. Kovalenko, R. Krasny, T. Kurtzman, T.S. Lee, S. LeGrand, P. Li, C. Lin, J. Liu, T. Luchko, R. Luo, V. Man, D.J. Mermelstein, K.M. Merz, Y. Miao, G. Monard, C. Nguyen, H. Nguyen, A. Onufriev, F. Pan, R. Qi, D.R. Roe, A. Roitberg, C. Sagui, S. Schott-Verdugo, J. Shen, C.L. Simmerling, J. Smith, J. Swails, R.C. Walker, J. Wang, H. Wei, L. Wilson, R.M. Wolf, X. Wu, L. Xiao, Y. Xiong, D.M. York and P.A. Kollman. AMBER 2019. *University of California, San Francisco* **2019**.

(7) Case, D. A.; Cheatham III, T. E.; Darden, T.; Gohlke, H.; Luo, R.; Merz Jr., K. M.; Onufriev, A.; Simmerling, C.; Wang, B.; Woods, R. J. The Amber biomolecular simulation programs. *J. Comput. Chem.* **2005**, *26*, 1668-1688.

(8) Salomon-Ferrer, R.; Case, D. A.; Walker, R. C. An overview of the Amber biomolecular simulation package. *WIREs Comput. Mol. Sci.* **2013**, *3*, 198-210.

(9) Word, J. M.; Lovell, S. C.; Richardson, J. S.; Richardson, D. C. Asparagine and glutamine: using hydrogen atom contacts in the choice of side-chain amide orientation. *J. Mol. Biol.* **1999**, *285*, 1735-1747.

(10) Loll, B.; Kern, J.; Saenger, W.; Zouni, A.; Biesiadka, J. Towards complete cofactor arrangement in the 3.0 Å resolution structure of photosystem II. *Nature* **2005**, *438*, 1040-1044.

(11) Guskov, A.; Kern, J.; Gabdulkhakov, A.; Broser, M.; Zouni, A.; Saenger, W. Cyanobacterial photosystem II at 2.9-Å resolution and the role of quinones, lipids, channels and chloride. *Nat. Struct. Mol. Biol.* **2009**, *16*, 334-342.

(12) The PyMOL Molecular Graphics System, Version 2.0; Schrödinger, LLC

(13) Beglov, D.; Roux, B. An Integral Equation To Describe the Solvation of Polar Molecules in Liquid Water. *J. Phys. Chem. B* **1997**, *101*, 7821-7826.

(14) Kovalenko, A.; Hirata, F. Three-dimensional density profiles of water in contact with a solute of arbitrary shape: a RISM approach. *Chem. Phys. Lett.* **1998**, *290*, 237-244.

(15) Imai, T.; Hiraoka, R.; Kovalenko, A.; Hirata, F. Water Molecules in a Protein Cavity Detected by a Statistical−Mechanical Theory. *J. Am. Chem. Soc.* **2005**, *127*, 15334-15335.

(16) Imai, T.; Hiraoka, R.; Kovalenko, A.; Hirata, F. Locating missing water molecules in protein cavities by the three-dimensional reference interaction site model theory of molecular solvation. *Proteins: Struct., Funct., Bioinf.* **2007**, *66*, 804-813.

(17) Luchko, T.; Gusarov, S.; Roe, D. R.; Simmerling, C.; Case, D. A.; Tuszynski, J.; Kovalenko, A. Three-Dimensional Molecular Theory of Solvation Coupled with Molecular Dynamics in Amber. *J. Chem. Theory Comput.* **2010**, *6*, 607-624.

(18) Yu, H. A.; Roux, B.; Karplus, M. Solvation thermodynamics: An approach from analytic temperature derivatives. *J. Chem. Phys.* **1990**, *92*, 5020-5033.

(19) Berendsen, H.; Grigera, J.; Straatsma, T. The missing term in effective pair potentials. *J. Phys. Chem.* **1987**, *91*, 6269-6271.

(20) Sindhikara, D. J.; Yoshida, N.; Hirata, F. Placevent: An algorithm for prediction of explicit solvent atom distribution—Application to HIV-1 protease and F-ATP synthase. *J. Comput. Chem.* **2012**, *33*, 1536-1543.

(21) Sindhikara, D. J.; Hirata, F. Analysis of Biomolecular Solvation Sites by 3D-RISM Theory. *J. Phys. Chem. B* **2013**, *117*, 6718-6723.

(22) Phongphanphanee, S.; Yoshida, N.; Hirata, F. On the Proton Exclusion of Aquaporins: A Statistical Mechanics Study. *J. Am. Chem. Soc.* **2008**, *130*, 1540-1541.

(23) Phongphanphanee, S.; Rungrotmongkol, T.; Yoshida, N.; Hannongbua, S.; Hirata, F. Proton Transport through the Influenza A M2 Channel: Three-Dimensional Reference Interaction Site Model Study. *J. Am. Chem. Soc.* **2010**, *132*, 9782-9788.

(24) Schott-Verdugo, S.; Gohlke, H. PACKMOL-Memgen: A Simple-To-Use, Generalized Workflow for Membrane-Protein–Lipid-Bilayer System Building. *J. Chem. Inf. Model.* **2019**, *59*, 2522-2528.

(25) Martinez, L.; Andrade, R.; Birgin, E. G.; Martinez, J. M. PACKMOL: a package for building initial configurations for molecular dynamics simulations. *J. Comput. Chem.* **2009**, *30*, 2157-2164.

(26) Bayly, C. I.; Cieplak, P.; Cornell, W.; Kollman, P. A. A well-behaved electrostatic potential based method using charge restraints for deriving atomic charges: the RESP model. *J. Phys. Chem.* **1993**, *97*, 10269-10280.

(27) Duan, Y.; Wu, C.; Chowdhury, S.; Lee, M. C.; Xiong, G.; Zhang, W.; Yang, R.; Cieplak, P.; Luo, R.; Lee, T.; Caldwell, J.; Wang, J.; Kollman, P. A Point-Charge Force Field for Molecular Mechanics Simulations of Proteins Based on Condensed-Phase Quantum Mechanical Calculations. *J. Comput. Chem.* **2003**, *24*, 1999-2012.
(28) Becke, A. D. Density-functional thermochemistry. III. The role of exact exchange. *J. Chem. Phys.* **1993**, *98*, 5648-5652.
(29) Becke, A. D. A new mixing of Hartree–Fock and local density-functional theories. *J. Chem. Phys.* **1993**, *98*, 1372-1377.
(30) Sigfridsson, E.; Ryde, U. Comparison of methods for deriving atomic charges from the electrostatic potential and moments. *J. Comput. Chem.* **1998**, *19*, 377-395.
(31) Cornell, W. D.; Cieplak, P.; Bayly, C. I.; Gould, I. R.; Merz, K. M.; Ferguson, D. M.; Spellmeyer, D. C.; Fox, T.; Caldwell, J. W.; Kollman, P. A. A Second Generation Force Field for the Simulation of Proteins, Nucleic Acids, and Organic Molecules. *J. Am. Chem. Soc.* **1995**, *117*, 5179-5197.
(32) Neese, F. Software Update: the ORCA Program System, Version 4.0. *WIREs Comput. Mol. Sci.* **2018**, *8*, e1327.
(33) Lu, T.; Chen, F. Multiwfn: A multifunctional wavefunction analyzer. *J. Comput. Chem.* **2012**, *33*, 580-592.
(34) Li, P.; Merz, K. M. Metal Ion Modeling Using Classical Mechanics. *Chem. Rev.* **2017**, *117*, 1564-1686.
(35) Maier, J. A.; Martinez, C.; Kasavajhala, K.; Wickstrom, L.; Hauser, K. E.; Simmerling, C. ff14SB: Improving the Accuracy of Protein Side Chain and Backbone Parameters from ff99SB. *J. Chem. Theory Comput.* **2015**, *11*, 3696-3713.
(36) Jorgensen, W. L.; Chandrasekhar, J.; Madura, J. D.; Impey, R. W.; Klein, M. L. Comparison of simple potential functions for simulating liquid water. *J. Chem. Phys.* **1983**, *79*, 926-935.
(37) Wang, J.; Wolf, R. M.; Caldwell, J. W.; Kollman, P. A.; Case, D. A. Development and testing of a general amber force field. *J. Comput. Chem.* **2004**, *25*, 1157-74.
(38) Dickson, C. J.; Madej, B. D.; Skjevik, Å. A.; Betz, R. M.; Teigen, K.; Gould, I. R.; Walker, R. C. Lipid14: The Amber Lipid Force Field. *J. Chem. Theory Comput.* **2014**, *10*, 865-879.
(39) Skjevik, Å. A.; Madej, B. D.; Walker, R. C.; Teigen, K. LIPID11: A Modular Framework for Lipid Simulations Using Amber. *J. Phys. Chem. B* **2012**, *116*, 11124-11136.
(40) Gounaris, K.; Whitford, D.; Barber, J. The effect of thylakoid lipids on an oxygen-evolving Photosystem II preparation. *FEBS Lett.* **1983**, *163*, 230-234.
(41) Guerra, F.; Siemers, M.; Mielack, C.; Bondar, A.-N. Dynamics of Long-Distance Hydrogen-Bond Networks in Photosystem II. *J. Phys. Chem. B* **2018**, *122*, 4625-4641.
(42) Sakashita, N.; Watanabe, H. C.; Ikeda, T.; Saito, K.; Ishikita, H. Origins of Water Molecules in the Photosystem II Crystal Structure. *Biochemistry* **2017**, *56*, 3049-3057.
(43) Ceccarelli, M.; Procacci, P.; Marchi, M. An ab initio force field for the cofactors of bacterial photosynthesis. *J. Comput. Chem.* **2003**, *24*, 129-142.
(44) Giammona, D. A. *Ph.D. thesis,University of California at Davis* **1984**.
(45) Li, P.; Roberts, B. P.; Chakravorty, D. K.; Merz, K. M. Rational Design of Particle Mesh Ewald Compatible Lennard-Jones Parameters for +2 Metal Cations in Explicit Solvent. *J. Chem. Theory Comput.* **2013**, *9*, 2733-2748.
(46) Li, P.; Song, L. F.; Merz, K. M. Parameterization of Highly Charged Metal Ions Using the 12-6-4 LJ-Type Nonbonded Model in Explicit Water. *J. Phys. Chem. B* **2015**, *119*, 883-895.
(47) Joung, I. S.; Cheatham, T. E. Molecular Dynamics Simulations of the Dynamic and Energetic Properties of Alkali and Halide Ions Using Water-Model-Specific Ion Parameters. *J. Phys. Chem. B* **2009**, *113*, 13279-13290.
(48) Joung, I. S.; Cheatham, T. E. Determination of Alkali and Halide Monovalent Ion Parameters for Use in Explicitly Solvated Biomolecular Simulations. *J. Phys. Chem. B* **2008**, *112*, 9020-9041.
(49) Essmann, U.; Perera, L.; Berkowitz, M. L.; Darden, T.; Lee, H.; Pedersen, L. G. A Smooth Particle Mesh Ewald Method. *J. Chem. Phys.* **1995**, *103*, 8577-8593.
(50) Loncharich, R. J.; Brooks, B. R.; Pastor, R. W. Langevin dynamics of peptides: The frictional dependence of isomerization rates of N-acetylalanyl-N′-methylamide. *Biopolymers* **1992**, *32*, 523-535.
(51) Ben-Shalom, I. Y.; Lin, C.; Kurtzman, T.; Walker, R. C.; Gilson, M. K. Simulating Water Exchange to Buried Binding Sites. *J. Chem. Theory Comput.* **2019**, *15*, 2684-2691.
(52) Berendsen, H. J. C.; Postma, J. P. M.; Gunsteren, W. F. v.; DiNola, A.; Haak, J. R. Molecular dynamics with coupling to an external bath. *J. Chem. Phys.* **1984**, *81*, 3684-3690.
(53) Ryckaert, J.-P.; Ciccotti, G.; Berendsen, H. J. C. Numerical integration of the cartesian equations of motion of a system with constraints: molecular dynamics of *n*-alkanes. *J. Comput. Phys.* **1977**, *23*, 327-341.
(54) Götz, A. W.; Williamson, M. J.; Xu, D.; Poole, D.; Le Grand, S.; Walker, R. C. Routine Microsecond Molecular Dynamics Simulations with AMBER on GPUs. 1. Generalized Born. *J. Chem. Theory Comput.* **2012**, *8*, 1542-1555.
(55) Salomon-Ferrer, R.; Götz, A. W.; Poole, D.; Le Grand, S.; Walker, R. C. Routine Microsecond Molecular Dynamics Simulations with AMBER on GPUs. 2. Explicit Solvent Particle Mesh Ewald. *J. Chem. Theory Comput.* **2013**, *9*, 3878-3888.
(56) Le Grand, S.; Götz, A. W.; Walker, R. C. SPFP: Speed without compromise—A mixed precision model for GPU accelerated molecular dynamics simulations. *Comput. Phys. Commun.* **2013**, *184*, 374-380.
(57) Roe, D. R.; Cheatham, T. E. PTRAJ and CPPTRAJ: Software for Processing and Analysis of Molecular Dynamics Trajectory Data. *J. Chem. Theory Comput.* **2013**, *9*, 3084-3095.

(58) Roe, D. R.; Cheatham III, T. E. Parallelization of CPPTRAJ enables large scale analysis of molecular dynamics trajectory data. *J. Comput. Chem.* **2018**, *39*, 2110-2117.
(59) Humphrey, W.; Dalke, A.; Schulten, K. VMD: Visual molecular dynamics. *J. Mol. Graphics* **1996**, *14*, 33-38.
